# Nanocarriers for Biomedicine: From Lipid Formulations to Inorganic and Hybrid Nanoparticles

**DOI:** 10.3390/ijms22137055

**Published:** 2021-06-30

**Authors:** Ruslan Kashapov, Alsu Ibragimova, Rais Pavlov, Dinar Gabdrakhmanov, Nadezda Kashapova, Evgenia Burilova, Lucia Zakharova, Oleg Sinyashin

**Affiliations:** A.E. Arbuzov Institute of Organic and Physical Chemistry, FRC Kazan Scientific Center of RAS, Arbuzov Street 8, 420088 Kazan, Russia; alsu_i@mail.ru (A.I.); rais.pavlov@iopc.ru (R.P.); nemezc1988@yandex.ru (D.G.); kashapova.nadya@gmail.com (N.K.); burilovajen07@mail.ru (E.B.); lucia@iopc.ru (L.Z.); oleg@iopc.ru (O.S.)

**Keywords:** drug delivery, liposome, non-covalent modification, surfactant, peptide, macrocycle, mesoporous silica, hybrid nanocarriers, cerasome

## Abstract

Encapsulation of cargoes in nanocontainers is widely used in different fields to solve the problems of their solubility, homogeneity, stability, protection from unwanted chemical and biological destructive effects, and functional activity improvement. This approach is of special importance in biomedicine, since this makes it possible to reduce the limitations of drug delivery related to the toxicity and side effects of therapeutics, their low bioavailability and biocompatibility. This review highlights current progress in the use of lipid systems to deliver active substances to the human body. Various lipid compositions modified with amphiphilic open-chain and macrocyclic compounds, peptide molecules and alternative target ligands are discussed. Liposome modification also evolves by creating new hybrid structures consisting of organic and inorganic parts. Such nanohybrid platforms include cerasomes, which are considered as alternative nanocarriers allowing to reduce inherent limitations of lipid nanoparticles. Compositions based on mesoporous silica are beginning to acquire no less relevance due to their unique features, such as advanced porous properties, well-proven drug delivery efficiency and their versatility for creating highly efficient nanomaterials. The types of silica nanoparticles, their efficacy in biomedical applications and hybrid inorganic-polymer platforms are the subject of discussion in this review, with current challenges emphasized.

## 1. Introduction

One of the ways to improve the effectiveness of medical therapies is the use of drug delivery systems. This makes it possible to increase bioavailability and biocompatibility of drugs, to optimize their release profile, overcome biological barriers, and control targeting properties, cellular uptake and intracellular trafficking [1,2,3]. Of particular interest is the delivery of anticancer drugs that can, in the future, reduce the severity of disease and mortality rate. The chemotherapy drugs used today for the treatment of tumors, such as 5-fluorouracil, methotrexate and doxorubicin (DOX), do efficiently suppress tumors, but also cause concomitant tissue damage, which leads to the development of adverse reactions comparable in severity to the underlying disease. In this concern, the nanosystems capable of shielding a chemotherapy drug from interacting with healthy tissues and their release after penetrating into tumor cells, are of great interest. Despite numerous publications on the topic, this problem has not yet been fully resolved, and further progress in this area is obviously related to a rational design of nanocarriers with controlled transport properties, morphology and size, taking into account the behavior in biological media and mechanisms of penetration into cells.

To construct drug carriers, numerous strategies are developed, in which different factors should be taken into account, including size characteristics, composition of formulations, nature and therapeutic indications of the drug, and a route of administration. A powerful tool for engineering the formulated medicines is their conjugation with functional ligands, thereby producing prodrugs with beneficial characteristics. For this purpose, polyethylene glycol (PEG), peptides and amphiphilic molecules are used [4,5,6,7,8], which allow modifying pharmacokinetics, toxicity, targeting properties, stimuli responsibility and other effects. Alternatively, encapsulation techniques are used, which provide effective facilities for the design of drug delivery systems. Importantly, the combination of these approaches may be successfully applied, resulting in synergic effect. Encapsulation strategies address different techniques and involve numerous types of systems composed of a variety of materials, which can be basically divided in organic and inorganic carriers. In turn, the two main families of organic systems are those based on amphiphilic (lipid and surfactant) [9,10,11] and polymeric [12,13] compounds. Both these groups provide marked advances in efficacy of loaded drugs due to optimizing their circulation and stability in bioenvironment. Essential feature of organic formulations is soft nature of the systems that allow for tailoring of morphology and shape characteristics, thereby providing the diversity of forms and sub-types of nanocarriers, including liposomes, solid lipid nanoparticles, nanostructural lipid particles, nanoemulsions, cubosomes, polymersomes, etc. [14,15]. Inorganic nanoparticles, e.g., metal oxides, gold and silica particles, fullerenes, quantum dots, etc., provide effective and versatile platform for the fabrication of drug delivery vehicles and diagnostic imaging agents [16,17,18]. These materials demonstrate exclusive optical, magnetic and electric properties in combination with enhanced loading capacity, mechanical stability, easy fabrication, controlled size and/or pore characteristics and other beneficial features, which can be tailored through proper choice of synthetic and functionalization techniques. Meanwhile, the highest biomedicine potential in terms of efficacy of therapy, safety, targeted delivery, triggered release, reduced toxicity and side effect can be offered by hybrid nanocarriers [19,20,21,22]. The current trends towards the development of hybrid formulations allow for attaining the synergy of beneficial properties of organic/inorganic [18], polymer/inorganic [19], lipid/inorganic [20], lipid/polymer [21] systems, etc.

**Figure 1:**
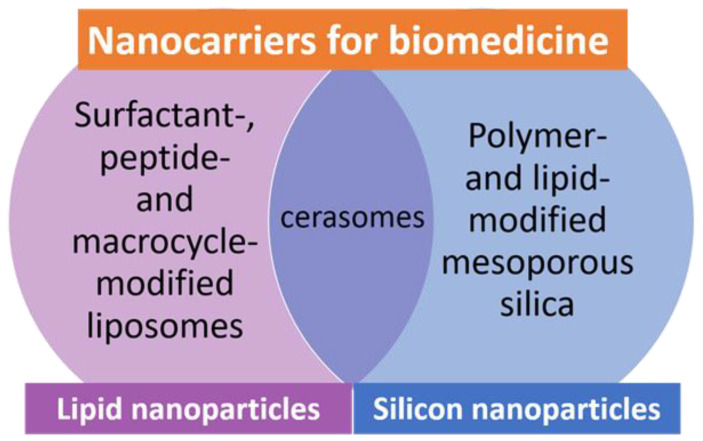
Main types of lipid- and silicon-containing nanoparticles, comprising many kinds of nanocarriers that can be used to achieve the best medical performance.

The urgency of this review is motivated by an intensively developed sphere of drug delivery, which is reflected in sharp growth of publications on this theme. High interest of researchers in the design of different types of nanocontainers occurs, with a clear trend towards multifunctional and complicated formulations observed. Meanwhile, there are a number of recent comprehensive reviews in different specific aspects, in which drug delivery systems are classified based on the chemical nature and prescription of a medicine, the type of nanocarriers [9], the target site [3,23,24] and the administration route [25,26]. Alternatively, based on the above analysis, two distinct families of nanocarriers, namely, lipid formulations and silicon-containing nanoparticles (Figure 1), as well as intermediary cerasomes, have been chosen herein, with a focus on recent publications covering the drug encapsulation technique and emphasizing the aspects insufficiently highlighted elsewhere, e.g., development of hybrid formulations via non-covalent modification of nanocarriers aimed at the improving of their stability, targeting effects, multicentered drug loading and toxicity profile. First, liposomal formulations are discussed, with main attention paid to those non-covalently modified with surfactants, peptide moieties, macrocycles and other ligands that may offer targeting properties, morphological diversity, additional binding sites for drugs, etc. Further, mesoporous silica nanoparticles are reviewed, with those modified by polymers emphasized. Various liposomal forms containing inorganic fragments, namely lipid-modified mesoporous nanoparticles and cerasomes are also considered. Such structure of the review may allow researchers to receive comparative analysis of these nanocarriers and their potential applications for different drugs and administration pathways.

## 2. Lipid Formulations: Modification with Surfactants, Peptides, and Macrocycles

### 2.1. Lipid Nanocarriers Modified with Amphiphilic Molecules

Liposomes are currently the most efficient and traditional drug delivery systems [1,2,9]. They are composed of phospholipids, which are the structural basis of all cell membranes, providing a good penetration of liposomes to cells. The advantage of such nanocarriers is their versatility, biocompatibility and biodegradability. In addition, liposomes are relatively easy to obtain under laboratory and industrial conditions. For these reasons, many FDA-approved liposomal preparations are available as anti-cancer and anti-fungal drugs, and even more potential formulations are on clinical trials [9]. It is known that instability under physiological conditions makes it difficult to use naked liposomes as targeted delivery systems without prior modification. Therefore, serious efforts have been undertaken to develop further generations of liposomal carriers, including stealth (PEGylated), antibody-conjugated liposomes, etc. [1,2]. Meanwhile, the problem of instability in bioenvironment may be reduced by inducing the charge on the surface of liposomes. This in turn may increase their ability to overcome biological barriers, thereby ensuring the loading and delivery of anionic forms of drugs and gene material mediated by cationic lipid carriers [27,28]. 

Promising strategy is the functionalization of liposomes with surfactants, which allows for controlling the size, shape and charge characteristics of vesicular nanocarriers, improving drug loading, overcoming the biological barriers, etc. [29,30,31,32,33,34,35,36]. Key factors that should be considered upon the modification are the nature of surfactant head group, hydrophobicity and presence of unsaturated C-C bonds in alkyl chains, and lipid/surfactant ratio [29]. It was demonstrated that surfactants are capable of irreversible integration with lipid bilayer far beyond their critical micellar concentration due to lateral interactions [30], thereby markedly changing surface characteristics of liposomes. Surfactant-modified vesicles exhibit essentially improved physicochemical properties and functionality, which in some cases resulted in the isolation of special types of flexible or elastic (deformable) vehicles with enhanced skin permeability, the so-called transferosomes [26,31,32,33], and alternative carriers, niosomes [35].

**Figure 2:**
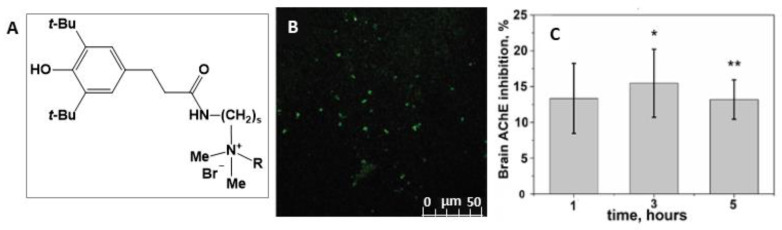
Structures of sterically hindered phenol containing quaternary ammonium moiety (SHP-s-R), where s = 2, 3; R=CH_2_Ph (SHP-2-Bn); R=C_n_H_2n+1_, n = 8, 10, 12, 16 (SHP-2-R) (**A**); Section of rat cerebral cortex (**B**); Determination of brain AChE inhibition level in vivo (**C**) after intranasal administration of PC/SHP-2-Bn/SHP-2-16 nanoparticles. * *p* = 0.028 and ** *p* = 0.004 indicate differences by Mann–Whitney test. Reprinted with permission from [40]. Copyright 2020 Royal Society of Chemistry.

Research activity of our group focuses on self-assembling amphiphilic systems, with their design, aggregation ability and functionality as drug delivery systems emphasized [2,37,38]. Recently, much attention has been paid to non-covalent modification of liposomes with surfactants [37,38,39,40], with cationic liposomes attracting special interest [41,42,43,44,45,46,47]. In [39,40] lipid nanoformulations based on L-α-phosphatidylcholine (PC), cholesterol (CHO) and hybrid amphiphilic ligands–quaternary ammonium salts with a sterically hindered phenolic (SHP) moiety with benzyl (SHP-2-Bn) and alkyl tails (SHP-2-R, where R=C_n_H_2n+1_, n = 8, 10, 12, 16) were obtained and characterized. Cationic liposomes based on PC with a diameter of 100 nm, modified with benzyl derivatives of SHP, exhibit more pronounced antioxidant activity than individual SHP-n-Bn. It has been found [39] that the stability, encapsulation efficacy, loading capacity and release from liposomes of a model dye, Rhodamine B, depend on the structure of SHP-n-Bn. Cationic liposomes based on PC and SHP-2-Bn show good stability in time (1 year) and sustained release (>65 h). A decrease in the antioxidant activity of SHP-n-Bn-liposomes with an increase in the length of the spacer has been shown. Alkyl-conjugated SHPs exhibit inhibition activity against acetylcholinesterase (AChE) and butyrylcholinesterase in combination with antioxidant properties, which allowed them to be considered as candidates for drugs against Alzheimer’s disease. Based on the data on self-assembly, their lipophilicity has been optimized and the relationship between biological activity and toxicity has been evaluated, which served as fundamentals for the development of multitargeted liposomal formulations [40]. Intranasal (in vivo) administration of PC/SHP-2-Bn/SHP-2-16 liposomes to rats (dose SHP-2-16 8 mg/kg) showed their ability to overcome the blood-brain barrier (BBB), providing the inhibition of AChE in the brain (up to 15.46%) (Figure 2). This makes it possible to develop potential effective drugs with a prospect in the treatment of Alzheimer’s disease.

Cationic liposomes were successfully used as drug carriers for overcoming the BBB, both via intranasal and intravenous administration [41,43]. For this purpose, liposomes based on PC were modified with double-chained surfactant dihexadecylmethylhydroxyethylammonium bromide [41]. This amphiphilic compound showed spontaneous self-assembly under low concentration of 0.01 mM, along with high solubilization and antimicrobial effects. Cationic liposomes of 100 nm bearing zeta potential of +25 mV were stable in time and characterized with high encapsulation efficiency of 90% toward encapsulated molecules, including AChE acetylcholinesterase reactivator pralidoxime chloride (2-PAM). In this study, 12% reactivation of brain AChE poisoned by organophosphorus pesticide, paraoxon, was achieved through intranasal administration of 2-PAM-loaded cationic liposomes, which appeared more advanced compared to intravenous route. 

Cationic liposomes modified with hydroxyethylated gemini surfactant 16-6-16(OH) were designed in [43], based on fundamental data on self-assembly and functional activity of the geminis [48,49]. Liposome composition was optimized (lipid/surfactant molar ratio = 35:1), so that delicate balance was attained between colloidal stability and toxicity caused by cationic surface charge. These 2-PAM-loaded liposomes were able to overcome BBB and reactivate phosphorylated AChE by 27% after intravenous administration, with no hemagglutination observed. Generally, toxicity of cationic formulation is one of the serious limitations, which require special precautions in every case. It was shown [50] that the increase of positive charge on the surface of the liposomes contributes to their greater penetration through the nasal epithelium, thereby ensuring a longer residence time for liposomal formulations in the nasal cavity. However, given the information that this is accompanied by increase in toxicity, the modification of liposomal formulations with biocompatible cationic polysaccharides, such as N-trimethyl chitosan or chitosan was used [51], with the former demonstrating superior effects over the latter in terms of biocompatibility and water solubility. 

Today, mitochondrion is recognized as a key target in therapy of a large variety of serious diseases, including diabetes, neurological and metabolic disorders, cancer, and others [3,52,53,54,55]. Mitochondria biomedicine has developed progressively as a special research field focusing on the design of mitochondria-tropic formulated drugs. In this regard, positively charged carriers are of particular interest due to their electrostatic affinity toward mitochondrion membranes bearing high negative potential. The most effective mitochondria-targeted ligand is the lipophilic triphenylphosphonium (TPP) cation that can be conjugated with different platforms, including liposomal vehicles [52,53,54,55]. In our recent publications [56,57], self-assembly and solubilization capacities of homological series of TPP surfactants have been evaluated with the aim of engineering of micellar nanocontainers and gene carriers. Further, non-covalent strategy was evolved for the design of mitochondria-targeted liposomes modified with TPP surfactants [42]. Considering that both the surface charge and toxic effect depend on alkyl chain length of surfactants, liposomal composition was optimized to ensure the balance between the stability, loading capacity and safety of formulations. Confocal microscopy study testified that Rhodamine B-loaded TPP-modified liposomes possess higher colocalization with the mitochondria of pancreatic tumor cells compared to unmodified formulations.

**Figure 3:**
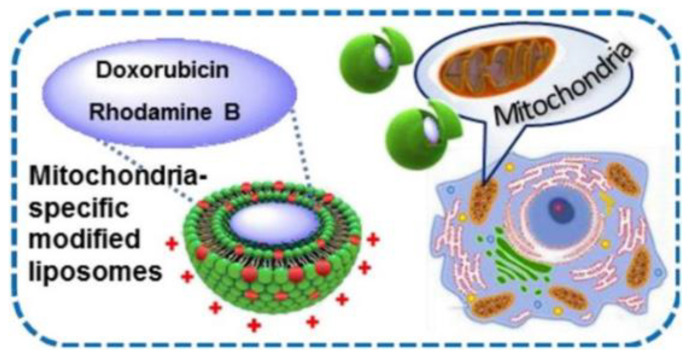
Schematic representation of mitochondria-specific modified liposomes. Reprinted with permission from [47]. Copyright 2021 Elsevier.

Given the information available on physicochemical and self-assembly behavior of two homological series of cationic surfactants with phosphonium and imidazolium (Im) head groups [56,57,58,59,60], an idea appeared to test whether amphiphilic imidazolium cation demonstrates the similar mitochondria-targeting effect as TPP analogs. For this purpose, liposomal formulations based on 1,2-dipalmitoyl-sn-glycero-3-phosphocholine (DPPC) DPPC were subjected to non-covalent modification with TPP- and Im-based surfactants with a tetradecyl hydrocarbon tails [47]. It has been shown that the obtained liposomes have a high degree of encapsulation efficiency toward Rhodamine B and DOX, as well as a significant positive charge, due to which their high shelf-life has been achieved (more than 4 months). Modified liposomes loaded with DOX accumulate in large amounts in duodenal adenocarcinoma and lung adenocarcinoma tumor cells, causing dose-dependent apoptosis. Importantly, similar to TPP cation, Im-based ligand is able of imparting the mitochondria-targeting to the nanocontainers (Figure 3), which was proven by confocal microscopy assays exemplified by A-549 and HuTu 80 cell lines. In the latter case, even higher Pearson coefficient occurs for Im versus TPP-based ligand.

Im-modified liposomes exhibit excellent long-term stability and high loading capacity toward a variety of drugs and spectral probes [44,45,46]. The authors of [44] obtained 70–100 nm sized liposomal formulations based on DPPC and Im-based amphiphiles with various length of hydrophobic tail for the encapsulation of a drug metronidazole. It has been shown that the modification of DPPC liposomes with such cationic amphiphiles resulted in zeta potential increase from +3 mV to +45–70 mV and improved the particle stability for a long time (more than 6 months, while unmodified liposomes decompose after 2 weeks). Notably, hybrid liposomal formulations composed of octadecyl derivative have the highest value of encapsulation efficiency of 75% for metronidazole. 

Two types of nanocontainers, micellar and liposomal, were prepared in [45] for conjugated derivative of pteridine and benzimidazole with antitumor activity. For micellar systems, an increase in solubility of the hydrophobic bioactive compound occurs in the series Tween 80 < CTAB < Im-16 surfactants. Optimized DPPC-based liposomes with lipid/Im-16 molar ratio of 50:1 exhibit long-term stability, size of 90 nm, low PdI ≤0.1 and prolonged release profile. Liposomal bioactive formulations show high cytotoxic activity toward M-HeLa cell line comparable with DOX, with a marked selective effect observed (cytotoxicity toward Chang Liver cell line was 37-fold lower).

DPPC-based liposomal carriers modified with a hydroxyethylated imidazolium surfactant with hexadecyl tail (Im-16-OH) were loaded with two hydrophilic drugs, antibiotic chloramphenicol and anticancer drug cisplatin [46]. Encapsulation techniques allowed for a 4-fold decrease the hemolytic activity of chloramphenicol and enhanced antitumor effect of cisplatin toward M-HeLa cells. Colocalization assay testified that decoration of liposomes with Im cation facilitated targeting properties of formulations to mitochondria compared to unmodified carriers. 

To fabricate the cationic liposomes different ligands and techniques are documented. The authors of [61] obtained cationic liposomes non-covalently modified with N,N,N-triethyl-N-(12-naphthoxydodecyl) ammonium bromide loaded with β-lactam antibiotic cefepime. It has been found that cefepime-loaded liposomes have a high inhibitory activity against *Escherichia coli* in vitro. The efficacy of cationic resveratrol-loaded liposomes modified with stearylamine in the treatment of hepatocellular carcinoma was described in [62]. The optimized liposomal formulation composed of soy lecithin, cholesterol and stearyl amine was a sphere of 145.78 ± 9.9 nm with zeta potential of +38.03 ± 9.12 mV and encapsulation efficacy (EE) of 78.14 ± 8.04%. The in vitro biocompatibility of such liposomes was confirmed by the absence of cytotoxicity against fibroblast L929 cell lines, as well as blood erythrocytes. In vitro cell culture assay showed an increased uptake of resveratrol-modified liposomes by hepatocellular carcinoma (HepG2) cells, which leads to a greater tumor cell killing ability compared with free resveratrol. In vivo pharmacokinetic and pharmacodynamic studies revealed selective accumulation of modified liposomal formulations in liver tumor tissue.

Alternatively, the synthesis of liposomes covalently modified with a quaternary ammonium compound for combination therapy against bacterial infection has been given in [63]. The quaternary ammonium compound ((11-mercaptoundecyl)-N,N,N-trimethylammonium bromide (MTAB)) has been attached to maleimide-functionalized liposomes via a thiol linker. Modified liposomes have been characterized by physicochemical methods. Their biological activity, in terms of antiadhesive activity and biofilm prevention in *Escherichia coli* has been evaluated. The results showed that MTAB-functionalized liposomes inhibit bacterial adhesion and biofilm formation, while reducing MTAB toxicity. 

Thus, an analysis of the literature on the modification of liposomes with surfactants has shown their efficacy in medicine as means of delivery and treatment of a number of diseases. Stabilization of liposomal formulations, targeting effects and overcoming of the biological barriers have been achieved by the presence of a charge on the surface of liposomes, which can be obtained by non-covalent and covalent modification with cationic surfactants. Non-covalent modification has a significant advantage over covalent modification in that there are no time-consuming multi-step organic synthesis procedures.

### 2.2. Peptides as Targeting Ligands for Lipid Formulations

Among other liposome modifications, a very promising way to enhance tumor specificity and therapeutic efficiency is to decorate liposomes with peptides. This can be easily done by anchoring of a peptide to the bilayer with a lipid-like moiety. So far, peptides used in liposomes have been conjugated to alkyl chains, hydrophobic amino acid sequences, lipids and pegylated lipids. According to the current review, the most common method of liposome modification in recent years is chemical conjugation of a peptide to a carboxy group or a thiolated peptide to maleimide group at the tip of the PEG chain (typically, 1,2-Distearoyl-sn-glycero-3-phosphorylethanolamine (DSPE) is used as an anchor). This approach is based on the need of liposomes to be long-circulating in the bloodstream, which is achieved by PEG addition, and to be able to selectively bind to desired sites such as tumor cells by providing a specific peptide modification.

Generally, a peptide attached to the liposomes serves a simple purpose of attachment of the liposome to a desired cell type, so that the drug carrier is concentrated and retained in tumor tissue to enhance the therapeutic effect and to lower systemic toxicity. Recently, the tripeptide Arg-Gly-Asp (RGD) was the most frequently used sequence for tumor targeting due to its short length and affinity to integrins, which are overexpressed on the surfaces of many types of cancer cells [64,65]. Among others, the cell penetrating peptides (CPPs) can be segmented to their own group; however, they lack cell specificity and are usually used in combination with active targeting peptides to help increase the cellular uptake [66]. Another group of peptides is targeting vascular endothelial growth factor receptor 2 (VEGFR-2), human epidermal growth factor receptor 2 (HER2) that are abundant in various cancer cell types and new tumor vasculature [67,68,69]. In the brain-targeted group, the most common targets are nicotinic acetylcholine receptor α7 (nAChR α7), neuropilin-1 and Interleukin-13 receptor subunit alpha-2 (IL-13Rα2) [66,70,71,72,73].

The most common way of liposome modification is addition of a functionalized pegylated lipid to the initial lipid mixture for thin film formation. Additionally, it was proposed in 2002 that liposomes can be functionalized by the post-insertion method since a hydrophobic anchor, present in such lipids as CHO or a lipid double chain moiety, is thermodynamically driven to be inserted into the liposome bilayer [74]. Since then, not much fundamental research has been devoted to this method, among which Kros’s group has varied different hydrophobic moieties, such as adamantane, hydrocarbon chains, CHO or lipid moiety, to compare their insertion efficiency and evaluate the ability of coiled coil-forming peptides to facilitate intermembrane fusion [75,76]. The work has shown that the hydrophobicity of the anchor can be crucial in some cases such as membrane fusion, where alkyl tails are not sufficient and a bulkier CHO moiety is required. Further, the nature of the hydrophobic anchor is important, as different anchors can induce different peptide conformations and thus can affect the peptide affinity to the target site.

**Figure 4:**
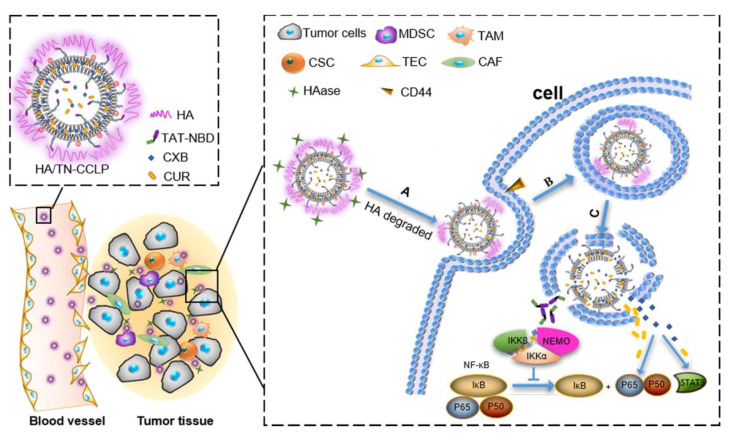
Schematic illustration of the in vivo fate of hyaluronic acid (HA) and TAT-NBD-modified liposomal system (HA/TN-CCLP). After intravenous injection, HA/TN-CCLP preferentially accumulate at the tumor tissues. (A) HA shell degraded or partially degraded by hyaluronidase (HAase), exposed TN-modified cationic liposome, and CD44 receptor promoted cellular uptake; (B, C) Endolysosomal escape. The released celecoxib (CXB), curcumin (CUR), and TAT-NBD peptide (TN) acted on NF-κB and STAT3. Abbreviations: MDSC—myeloid-derived suppressor cells, TAM—tumor-associated macrophages, CSC—cancer stem cells, TEC—tumor endothelial cells, CAF—cancer-associated fibroblasts. Reprinted with permission from [78]. Copyright 2019 American Chemical Society.

Nowadays the researcher is facing a lot of challenges when developing a liposomal formulation. The liposomes, ideally, must be able to accumulate at a desired location in the body, must be morphologically intact at any point before reaching the target, and once at the desired location, they must undergo cellular uptake and bypass lysosomal decomposition, and at this point, the contents should be released. Often one functional modification is not enough for a liposome, so peptide combinations are used. Such approach of combinational peptide liposome formulation has been done recently which allowed for glioma in vivo targeting and blood–brain barrier crossing by combining a cell-penetrating peptide R8 and transferrin [77]. Alternatively, dual-functioning peptides are being used, usually a cell-penetrating TAT peptide fused with a targeting peptide, for example, the NF-κB essential modulator binding peptides (NBD), since they are targeted at cell nuclei and must be able to traverse the cell membrane (Figure 4) [78]. Another recent example is CB5005, a sequence that contains a Leu-Ala rich hydrophobic domain for cell-penetrating properties and a targeting domain for nuclear localization [79]. The DP7-C sequence is one of such peptides, since it promotes both caveolin- and clathrin-dependent uptake as well as acting as an immune adjuvant, which makes the peptide an excellent ligand for mRNA delivery [80,81]. In fact, most promising peptides for nanocarrier modification are the ones that possess multiple aspects of activity, such as cell-penetrating, targeting, immune adjuvant, antimicrobial or stimuli-responsive properties.

This different class of peptides can be outlined as the ones designed to possess pH-dependent properties for targeting the low pH media of cancerous tissues. In this regard, a novel pHLIP peptide was developed and demonstrated for sensing acidic environment and trigger cargo release [82]. A H_6_L_9_ peptide designed to be pH-sensitive produces a negative charge for the liposome surface at pH 7.4, but in tumor environment (pH 6.3) it obtains a positive charge that makes liposomes adhere to the tumor cells, and in combination with the RGD motif the developed formulation was found to accumulate in C26-based colon tumor in vivo [83].

Another interesting approach to peptide design is retro inversion. A d-peptide was developed based on A7R peptide (ATWLPPR) that targets VEGFR2 and neuropilin-1 which is a retro inverso isomer ^D^A^D^T^D^W^D^L^D^P^D^P^D^R comprised of d-amino acids and it appeared to be more efficient for in vivo glioma targeting that the L-counterpart [84]. Same was found for the l- and d-versions of the CDX lipid that is targeted at nAChR α7 and the combination of ^D^CDX and ^D^A7R was able to target modified liposomes to glioma cells successfully in vitro and in vivo [70,71,85]. A ^D^T7 peptide designed to bind to transferrin was successfully screened on liposomes and showed very promising results in an in vivo hepatocellular carcinoma xenograft model [86].

The well-known RGD sequence is still being used due to its simplicity and useful integrin-targeting functionality to enhance cellular uptake [87,88]. Alternative α_v_β_3_ targeting sequences are being developed as well, which is important for the development of peptide-based formulation state of art. So, a mnRwr (lower case letters stand for d-amino acids) sequence was developed to target α_v_β_3_ integrin. The proposed motif has better immunocompatibility compared to the c(RGDyK), and was more efficient at binding to the α_v_β_3_ integrin. Overall, the novel mmRwr peptide was able to target glioma, angiogenesis, vasculogenic mimicry and produce a therapeutic effect when attached to DOX-loaded liposomes [89]. Another novel peptide targeting this protein is P1c that was shown to be efficient at targeting α_v_β_3_ expressing cancer cells [90]. A similar trend can be seen for cell-penetrating peptides. There are well-recognized sequences such as Pep-1, TAT that are classified as CPPs, which are often used in combination with targeting functions [91]. However, search for novel peptide sequences for cell penetration is still ongoing and novel motifs are introduced. For example, to significantly enhance docetaxel cytotoxicity on PEGylated liposomes in the ng/L range of drug, the RIPL peptide was introduced [92]. While similar to the CPPs, the TD sequence is also able to temporarily open the paracellular pathway and is best suited to enhance skin penetration for topical drug delivery [93]. A comparative study of six peptide ligands (Angiopep-2, T7, Peptide-22, c(RGDfK), D-SP5 and Pep-1) was conducted by Chen et al. to find that a combination of cRGD and Peptide-22 is very efficient at crossing BBB and blood-brain tumor barrier (BBTB) and targeting tumor cells in vivo on intracranial glioma-bearing mice [72]. PSP peptide is a novel penetratin-derived CPP incorporated in liposomes for treatment of fibrosarcoma in mice. In a combination with the NGR peptide, it was used for simultaneous targeting of CD13 expressing tumor cells as well as enhanced cellular uptake [94]. Other recently developed sequences such as RF [95] are being tested as CPPs. Another interesting sequence bearing both cell-penetrating properties and pH sensitivity is H_7_K(R_2_)_2_, developed by Zhao et al., which is transitioning from a hydrophobic state in normal pH to a hydrophilic state in acidic conditions, which destabilizes the modified liposomes for cargo release [96].

**Figure 5:**
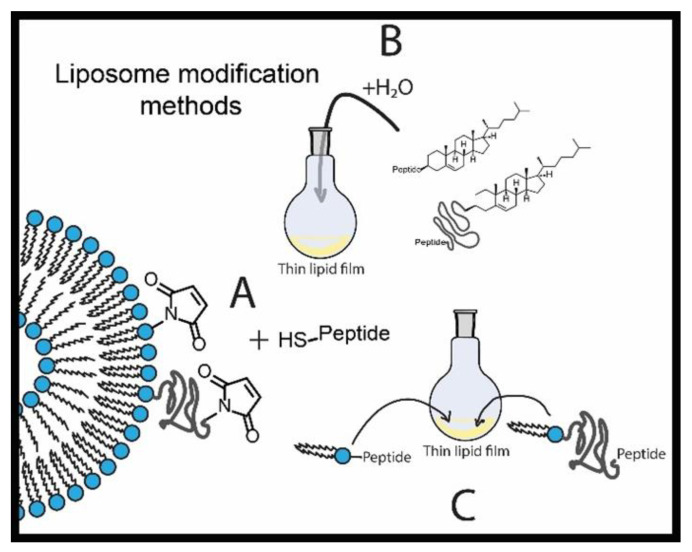
Different examples of liposome modification methods: (**A**) chemical conjugation to prepared liposomes (usually a fast reaction is applied such as thiol-maleimide coupling); (**B**) hydration with aqueous solutions of functionalized lipids (CHO shown as a sample anchoring moiety), or post-insertion; (**C**) incorporation of functionalized lipids to the initial lipid film.

Various methods of liposome functionalization with peptides are shown in Figure 5. The recent examples of novel successful peptides-based formulations are briefly listed in Table 1. Most of the work devoted to peptide-modified liposomes involves pegylated lipids capped with peptides for liposome modification. Although this approach does find success in screening both in vitro and in vivo, there is yet some critique towards application of long PEG chains capped with targeting ligands and towards the covalent methods of liposome modification, such as variable coupling yield, possibility of side reactions, and necessity of liposome purification after the insertion [97,98]. Furthermore, the general principle of targeted liposome preparation has some limitations. Traditionally, PEG-2000 is chosen as a stealth additive at 5–8 mol%. Later work has shown that there are practically no benefits for using more than 2 mol% of PEG-2000. It is also now known, contrary to the idea of the necessity of full liposome surface coverage, that while 2 mol% of PEG-350 is only covering about 17% of a 100-nm liposome, it is increasing the circulation time by almost 8 times. Protein binding and opsonization have been questioned as the crucial factors of circulation time prolongation, and alternative explanation has been proposed that PEG chains shield particles from coalescing and aggregating together in the bloodstream, keeping them as intact nanoscale particles [98]. Further work on the details of PEG application has shown that longer chains, starting with molecular weight of 2000 can obtain an undesirable coiled conformation that may hide the tip of the chain, where the targeting ligand is located, thus reducing the efficiency of targeting. It was shown that using short PEG-350 chains for stealth properties, and a little bit longer PEG-550 chains with targeting ligands is the optimal way to achieve efficient pegylated liposome uptake by the cells [97]. Despite the discovered problems associated with PEG, very few publications [68,99] in the recent years are utilizing the principles found by these fundamental papers. The researcher is encouraged to apply these data to design more advanced peptide-based liposomal formulations and to produce effort in liposome targeting technique optimization.

### 2.3. Lipid Formulations: Modification with Macrocycles (Cyclodextrins, Calixarenes and Porphyrins)

Despite the generally recognized benefits of liposomal carriers responsible for their wide application [110,111], there are several problems limiting the application of liposomes, such as stability issues, low drug entrapment, particle size control and short circulation half-life of vesicles [112]. The non-covalent inclusion of macrocycles in the liposomal structure helps to solve problems related to stability, inclusion of insoluble drugs into liposomes, drug-to-lipid ratio, target delivery and toxicity. In 1994 for the first time, the macrocyclic cyclodextrins (CDs) were entrapped into liposomes [113]. The inclusion complexes of dehydroepiandrosterone, retinol and retinoic acid were formed with 2-hydroxypropyl-β-CD (HP-β-CD) or β-CD polymers. Then, water-insoluble drugs in the form of CD-inclusion complexes were entrapped into the multilamellar liposomes by the dehydration–rehydration procedure. When loaded liposomes were exposed to blood plasma, almost all amount of CD remained inside the liposomes; however, the release of drugs was considerable, which is explained by the partial displacement of the drug from the CD cavity to the surface of lipid membrane. 

In [114], β-CD, methyl-β-CD (M-β-CD), HP-β-CD inclusion complexes containing poorly water-soluble drugs, sulfamerazine (SMR) and indomethacin (INM), in liposomes prepared from egg PC and CHO were obtained. According to DLS (dynamic light scattering) data, empty and drug-loaded liposomes were monodisperse small unilamellar vesicles. The stability of liposomes with embedded drugs and CDs was determined by the retention of calcein encapsulated in vesicle. The retention percentages for PC/CHO (3:1) liposomes containing SMR or INM were over 70% and 95%, respectively, indicating that the vesicles are stable even with the encapsulated drugs. Liposomes containing β-CD, HP-β-CD remained their integrity (retention of calcein was over 80%), while liposomes loaded with M-β-CD lost their stability within 48 h, which is explained by the ability of M-β-CD to form micelles with lipids. The drug per lipid concentration values were 308.98 and 10.14 mmol/mol for SMR and INM compositions, respectively. Entrapment value for INM in the presence of HP-β-CD with PC/CHO (3:1) liposomes were 43 times higher, in comparison with the pure liposomes. However, CDs did not improve the entrapment of SMR into liposomes due to high affinity of this hydrophobic drug for the lipid bilayer.

To increase the solubility and stability of hydrophobic drug CUR in blood, the CD/CUR inclusion complexes were entrapped into liposomes by the dehydration-rehydration vesicle method [115]. Drug-in-CD-in-liposome (DCL) formulations have significantly higher (up to ~2 times) loading efficiencies for CUR, compared to the conventional liposome. Herewith, liposomes containing HP-γ-CD complex demonstrate ~1.5 times higher loading efficiencies than those with the HP-β-CD complex. DCL approach increases CUR loading by ~23 times (depending on the lipid composition and CD used), resulting in higher solubility. CUR stability (at 0.01 and 0.05 mg/mL) in 80% (*v*/*v*) fetal bovine serum was evaluated at 37 °C. CUR stabilization was similar in hybrid (CUR -in-CD-in-liposome) and conventional liposomes. The entrapment of CUR in liposomes increases up to 56 times the amount of intact CUR in FBS after 24 h.

With the aim to improve the aqueous solubility of clove essential oil and its main component eugenol, DCL and double-loaded liposomes (DCL2), embedded with essential oil and eugenol were prepared at laboratory and large scale using a membrane contactor [116]. The sizes of the liposomes obtained by syringe injection and in the reactor were similar and well suited for further use in various industries (mean size from 199 ± 14 nm to 248 ± 29 nm with PDI from 0.150 ± 0.05 to 0.23 ± 0.06 and zeta potential from −13.5 ± 1.9 mV to −4.7 ± 0.5 mV). When eugenol was loaded into liposomes in the form of an inclusion complex with HP-β-CD, the loading rate increased by ~2 times for DCL and ~2.8 times for DCL2 compared to conventional liposomes without a macrocycle. For the first time, DCL formulations were freeze-dried and their stability was evaluated after reconstitution of lyophilized liposomes. In contrast to DCL2 systems, DCLs were stable in aqueous and lyophilized forms after 1 month storage at 4 °C. It was shown that HP-β-CD loaded into the aqueous core of the liposome in the case of DCL protects the system during freeze-drying [117].

The DCL approach was also used to improve the effectiveness of liposome loading with anethole (ANE), an essential oil component [118]. HP-β-CD/ANE inclusion complexes were encapsulated into liposomes (ACL), prepared from Phospholipon 90H or Lipoid S100. Moreover ANE-double-loaded liposomes (ACL2), where ANE is additionally added to the organic phase, were obtained. According to DLS and transmission electron microscopy studies, all the obtained vesicles had oligolamellar morphology. The DCL system significantly improved the loading of ANE. The values of loading rate were ~2 and ~3.5 times higher for ACL-90H and ACL2-90H, respectively, in comparison with ANE-loaded liposomes without HP-β-CD. Furthermore, the loading rates were higher for formulations with Lipoid S100 than Phospholipon 90H. It was also shown that the introduction of ANE in a complex with HP-β-CD into liposomes leads to an increase in photostability. UV radiation protection of ANE was improved by ~12, ~36, ~45 and ~67 times for ACL-90H, ACL2-90H, ACL-S100- and ACL2-S100, respectively, in comparison with free ANE in aqueous solution. All formulations except ACL2-S100 were stable after 15 months of storage at 4 0C and ensured the retention of more than 25% of ANE. Further study [119] of the stability of ANE-loaded liposomes showed that the presence of HP-β-CD protects liposomes prepared from hydrogenated (Phospholipon-90H and 80H) phospholipids during freeze drying. Freeze-dried ACL-90H were physically stable upon reconstitution in HP-ß-CD solutions and ensured the retention of ANE after 6 months of storage at 4 °C.

In the next work [120], the authors used the same approach to improve the physicochemical properties of quercetin (Quer). Liposomes based on Lipoid E80 had small diameter, narrow size distribution, and higher encapsulation efficiency of Quer as compared to formulations with Lipoid S100 and Phospholipon 90H. However, DCLs showed a lower encapsulation efficiency of Quer than conventional liposomes without the macrocycle (EE % for conventional liposomes and DCLs were 71.0 ± 2.0% and 27.9 ± 6.7%, respectively) due to the limited capacity of the aqueous core of liposomes to entrap CD/Quer inclusion complex. The photostability of Quer-loaded liposomes prepared with Lipoid E80 were higher than that with other lipids. Furthermore, the use of DCLs (sulfobutylether-β-CD/Quer in Lipoid E80 liposomes) provides additional photostability in comparison with conventional liposomes. Upon exposure of DCLs to 254-nm ultraviolet C during 72 h, more than 70% of Quer remained in the solution. The stability of Quer in simulated gastro-intestinal fluids was also higher with DCLs than conventional liposomes. All liposome formulations were physically stable and effectively retained Quer after 1 year of storage at 4 °C.

The encapsulation of nerolidol, a natural sesquiterpene, into Lipoid E80 liposomes as HP-β-CD/nerolidol complexes is described in [121]. The EE% values of nerolidol were ~97% and 93%, respectively, for HP-β-CD/nerolidol molar ratios of 1:1 and 2.5:1. However, high encapsulation efficiency (>97%) of nerolidol was also observed in conventional liposomes without HP-β-CD addition at different molar ratios of E80/nerolidol (100:1; 100:2.5; 100:5 or 100:10). The best loading capacity of nerolidol (70.76 ± 4.40%) was determined for DCL with E80/HP-β-CD/nerolidol molar ratio of 100:30:30. DCLs delayed the nerolidol release in comparison with CD/nerolidol inclusion complexes and conventional liposomes due to more barriers (macrocycle, lipid bilayer) to be overcome before release. It was shown that the release was more delayed with increasing CD/nerolidol molar ratios. Furthermore, DCLs formulations demonstrated higher photoprotection of nerolidol than conventional liposomes. The amount of nerolidol in liposomes and DCLs remained up to 99.99% and 98.67%, respectively, after storage for a year at 4 °C.

In order to improve the loading and delivery of paclitaxel (PTX), an anticancer drug, the double loaded PEGylated liposomes (DLPLs) containing PTX and PTX- dimethyl-β-CD complex were prepared by thin film hydration [122]. The solubility of pure PTX in water was 0.38 ± 0.05 μg/mL at 25 °C, and it was increased to 11.1 ± 0.22 mg/mL in a complex PTX/dimethyl-β-CD (1:20). According to DLS, the DLPLs were 162.8 ± 4.1 nm in diameter with PDI of 0.18 ± 0.004 and −5.6 ± 0.14 mV zeta potential. The obtaining of nanoscale particles was important for preventing of opsonization of the liposomes by phagocytes, enhanced permeability to tumor sites, and retention effect. The DLPLs entrapped 1.2 mg of PTX/mL, which was ~2 times higher than the loading efficiency of conventional PEGylated liposomes, PLs (0.58 mg PTX/mL). The in vitro drug release study showed that liposome formulations did not exhibit pH dependent release. Moreover, DLPLs had lower hemolysis and higher cytotoxicity on SKOV3 cells resulting in IC_50_ values ~4.2 and ~2.5 times lower than that for Taxol^®^ and PLs, respectively, after 48 h. In vivo pharmacokinetic study in rats revealed that PTX in DLPLs has a prolonged release profile, higher plasma concentration, and is more slowly eliminated from the circulation in comparison with Taxol^®^. In addition, the acute toxicity study with mice confirmed the safety of PTX-loaded liposomes.

The DCL approach was applied for temoporfin (meta-tetrakis(3-hydroxyphenyl)chlorin, mTHPC), a clinically approved photosensitizer, to improve the targeting of poorly soluble mTHPC [123]. The DCL formulations were prepared using various β-CD derivatives (HP-β-CD, M-β-CD and heptakis(2,3,6-tri-O-methyl)-β-CD (TM-β-CD)) and different amounts of mTHPC by thin film hydration. Using spectroscopic techniques, it was found that mTHPC molecules are mainly located in the inclusion complexes with β-CDs in the inner aqueous core of DCLs. The hydrodynamic diameter of DCLs varied from 125.7 ± 0.9 to 142.2 ± 0.8 nm with PDI between 0.037 ± 0.015 and 0.146 ± 0.040. The relatively strong negative surface charge of DCLs from (−36.7 mV) to (−39.0 mV) could prevent their aggregation ensuring colloidal stability. The DCL formulations with M- and trimethyl-β-CD as well as Foscan^®^ (commercial temoporfin formulation) remained stable more than 3 months. However, DCL based on HP-β-CD tended to degrade after 1.5 month of storage at 4 °C in the dark. The analysis of intracellular localization of mTHPC in HT29 human colon adenocarcinoma monolayer cells and mTHPC distribution in HT29 multicellular tumor spheroids after pre-treatment with DCLs showed that mTHPC distribution depend on CD in DCL formulations. DCL based on trimethyl-β-CD showed an almost homogeneous distribution of photosensitizer in tumor spheroids in contrast to DCL based on HP-β-CD and M-β-CD, as well as Foscan^®^ with a heterogeneous distribution. The difference is explained by a higher affinity of trimethyl-β-CD to mTHPC resulting in higher complex stability and, accordingly, a deeper delivery of mTHPC into tumor tissue after liposome destruction in the medium.

The entrapment of Estetrol (E4) hormone in HP-β-CD in liposomes has been applied with the aim to increase the passage through the BBB followed by accumulation of E4 in the brain as an approach for the treatment of hypoxic-ischemic encephalopathy in premature infants [124]. DCL system was 149 ± 6 nm in diameter with PDI of 0.06 ± 0.03 and 28 ± 2 mV zeta potential. Efficiency of E4 encapsulation for conventional liposomes and DCL systems varied from 3% to 10% and from 15% to 35%, respectively. In vitro E4 release studies showed a high drug release, reaching ~80% after 6 h and ~95% after 24 h for E4-loaded liposome and DCL. E4-loaded liposomes and DCL have been found to increase E4 penetration through BBB in comparison with free E4. The in vitro passage of E4-loaded liposomes and DCL through BBB model was ~5.0% after 2 h of contact, while BBB passage for free E4 was ~1.0%. The penetration increased to ~9.0% for E4-loaded liposomes and ~13.0% for DCL after 6 h, while in the case of free E4 there was no increase in BBB passage. The improvement of BBB passage was explained by the positive surface charge of the liposomes, which provides the interaction with negatively charged BBB membrane.

TM-β-CD and heptakis(2,3-di-O-acetyl)-β-CD (HDA-β-CD) were entrapped into unilamellar vesicles of 1-palmitoyl-2-oleoyl-sn-glycero-3-phosphocholine (POPC) [125]. The size of vesicles increased in proportion to the concentration of TM-β-CD and HDA-β-CD, while the size of the β-CD-loaded liposomal systems was not concentration-dependent. It was shown that functionalized TM-β-CD and HDA-β-CD, along with natural β-CD, stabilize the liposomal system due to the formation of hydrogen bonds with phospholipid head groups. According to the data of molecular dynamics modeling, these β-CDs do not have the tendency to penetrate the lipid bilayer.

**Figure 6:**
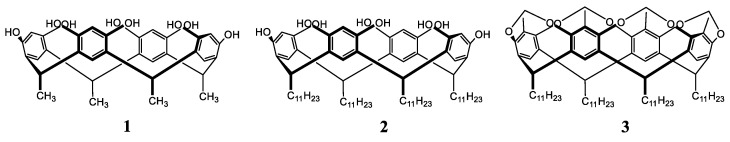
Chemical structures of calix[4]resorcinols 1, 2 and calix[4]resorcinol cavitand 3.

Calixarenes having hydroxyl groups, as well as CDs, are capable of forming hydrogen bonds with phospholipids, thereby stabilizing the liposomal system. In [126] calix[4]resorcinols 1, 2 and calix[4]resorcinol cavitand 3 (Figure 6) were embedded in liposomes prepared from POPC. The stability of liposomal systems was estimated by the spontaneous release of the entrapped 5(6)-carboxyfluorescein from liposomes. The introduction of 1 and 2 in the phospholipid bilayer decreases the rate of carboxyfluorescein release into the external environment by 2 times and liposome stabilization increases with an increase in the concentration of calix[4]resorcinols. The key role in the stabilizing effect of calix[4]resorcinols 1, 2 is related to the presence of hydroxyl groups in the structure capable of hydrogen bonding with the head groups of phospholipids. The embedment of cavitand 3 that does not contain hydroxyl groups, on the contrary, destabilizes the liposomal system. The first-order rate constant of CF release for liposomes loaded with cavitand 3 was higher than for pure liposomes.

**Figure 7:**
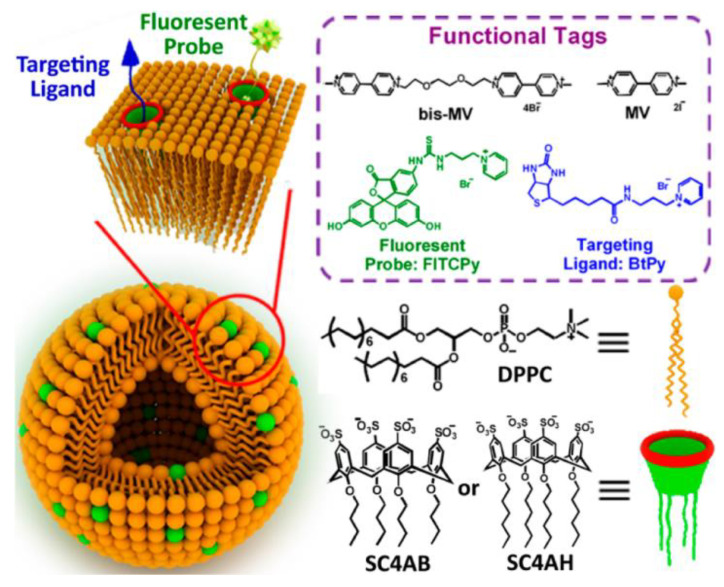
Schematic representation of a multifunctional liposomes, chemical structures of functional tags, DPPC, SC4AB and SC4AH. Reprinted with permission from [127]. Copyright 2015 American Chemical Society.

A stabilizing effect on DPPC liposomes was also achieved by calix[4]arenes SC4AB and SC4AH substituted with sulfonate groups at the upper rim and alkyl tails at the lower rim (Figure 7) [127]. The average size of liposomes embedded with calixarene was around 100 nm with addition of SC4AH and SC4AB to 5 and 10 mol%, respectively. A further increase in the calixarene content led to the destruction of large aggregates to particles with a size of 20–60 nm. The negative surface charge of DPPC-SC4AH and DPPC-SC4AB liposomes (−40.8 and −20.4 mV, respectively) contributed to a long-term storage of liposomal formulations up to 6 months due to electrostatic repulsion between adjacent bilayers, which prevents their aggregation. It was shown that the addition of bispyridinium salts of methyl viologen caused vesicle agglutination due to the guest-host interaction between viologens and calixarenes. Further, the liposome surface was modified with a fluorescent probe fluorescein isothiocyanate-conjugated pyridinium (FITCPy) and biotinylated pyridine as a targeting ligand. The functionalized liposomes were transferred to targeted cancer cells, demonstrating much better targeting activity toward MCF7 cancer cells, a type of human breast adenocarcinoma cells in comparison with free fluorescent probe and the nonfunctionalized mixed liposomes.

A series of calix[4]arenes bearing two imidazole and two ether substituents at the lower rim 1–3 were entrapped into POPC/CHO (7:3) large unilamellar vesicles (~0.2 µm) with a calixarene/lipid ratio of 1:500 [128]. The ability of 1–3 to transport copper (I) through the phospholipid membrane was studied by fluorescence spectroscopy using bathocuproin disulfonate. It was shown that calixarenes 2 and 3 transfer Cu^+^ more than twice as fast as 1, while the transport did not occur in the absence of calixarene in the liposomal system. The binding of Cu^+^ was due to the presence of several nitrogen atoms in the calixarene structure. The structural fragment of calixarene, 2-((4-(tert-butyl)phenoxy)methyl)-1-methyl-1H-imidazole, was not capable of transferring copper (I) across the membrane, which indicates the importance of preorganization of imidazole groups on the calixarene platform.

The embedment of disubstituted imidazole derivative of calix[4]arene into DPPC liposomes was also carried out to accelerate Suzuki-Miyaura coupling of 1-bromo-4-nitrobenzene with phenylboronic acid in water [129]. The entrapment of calixarene into multilamellar DPPC liposomes leads to their transformation into unilamellar ones, while the particle size before extrusion decreased from 600 ± 63 nm for pure DPPC vesicles to 60 ± 1 nm for calixarene-loaded liposomes at a calixarene-to-lipid molar ratio of 0.07. Calix[4]arene in a complex with Pd(OAc)_2_ loaded into DPPC liposomes exhibited higher catalytic activity in Suzuki–Miyaura coupling compared with pure Pd(OAc)_2_, calixarene-Pd(II) complexes or Pd(OAc)_2_ entrapped into DPPC liposomes without calixarene.

**Figure 8:**
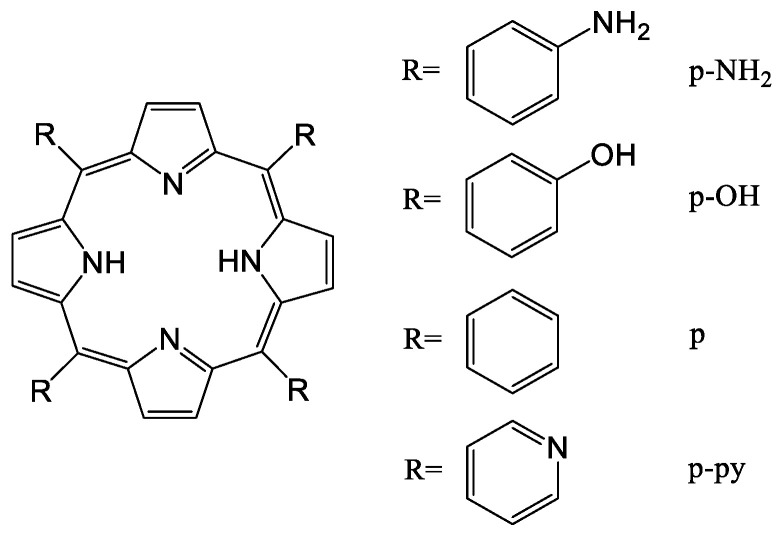
Chemical structures of porphyrins (p-NH_2_, p-OH, p, and p-py).

Other macrocyclic compounds, porphyrins, are also loaded in liposomal formulations in order to solve the problems limiting their application as photosensitizers, such as water solubility, delivery to the targeted area, internalization into tumor cells, biocompatibility, damage to healthy tissue upon irradiation, or insufficient generation of singlet oxygen for photodynamic therapy (PDT), etc. In order to enhance the delivery efficiency to tumor cells in [130], four different derivatives of porphyrin (p-NH_2_, p-OH, p and p-py) were entrapped in the lipid bilayer [131,132] of liposomes (Figure 8). Among the studied porphyrins, 5,10,15,20-tetrakis(4-hydroxyphenyl)porphyrin (p-OH) showed the best loading efficiency, and the p-OH-loaded liposomes revealed the highest toxicity to cancer cells under and without exposure to light. Additional coating of these liposomes with hyaluronic acid led to an increase in the affinity of the liposome for cancer cells due to binding of hyaluronic acid to MDA-MB-231 cells.

In [133], photosensitive liposomes were prepared, based on three different phospholipids (1-stearoyl-2-oleoyl-sn-glycero-3-phosphocholine (SOPC), 1,2-dioleoylsn-glycero-3-phosphocholine (DOPC) or 1-stearoyl-2-linoleoyl-sn-glycero-3-phosphocholine (SLPC)) and then loaded with photosensitizers (5,10,15,20-tetrakis(m-hydroxyphenyl)porphyrin, verteporfin, or pheophorbide a). Comparative analysis of the efficiency of the lipid membrane destruction upon irradiation followed by the release of porphyrin showed that DOPC liposomal formulation containing porphyrin with hydroxyphenyl groups was the most effective one. In DOPC liposomes, the release of this macrocycle reached 40% after 6 h of irradiation at a low dose of 2 mW/cm^2^.

The therapeutic efficacy of the combined use of PDT and liposomes loaded with plant-derived porphyrin-related macrocycle, pyropheophorbide acid (PPa), as photosensitizer was shown in [134]. Irradiated with a laser at 690 nm, PPa was activated for fluorescence imaging and PDT of cancer. In vitro and in vivo experiments demonstrated that PPa-loaded liposomes significantly inhibited the tumor growth under laser irradiation compared to other control groups. Moreover, liposomes embedded with PPa showed long-term circulation in blood and a high rate of accumulation in tumor after intravenous injection in mice.

The photosensitizer Chlorin e6 (Ce6) and low-molecular citrus pectin (LCP) as antagonist of galectin-3 were entrapped into liposomes of dialkyl PC lipids modified by 1,2-dioleoyl-3-trimethylammonium-propane and 1,2-distearoyl-sn-glycero-3-phosphoethanolamine-N-poly(ethyleneglycol)-2000 with a hydrodynamic diameter of ~130 nm [135]. Under irradiation, due to the presence of Ce6 in the lipid bilayer, the liposome was destroyed by the oxidation of the phospholipid membrane, and LCP was released from the hydrophilic core into the external environment. The studied effects of formed liposomes on A375 cells and tumor-bearing nude mice demonstrated that the released LCP moved to the cytoplasm, where it inhibits the activity of galectin-3, which enhanced the PDT effect of Ce6 in melanoma treatment, inhibited the ability to invade tumor cells and enhanced the immune effect of lymphocytes.

**Figure 9:**
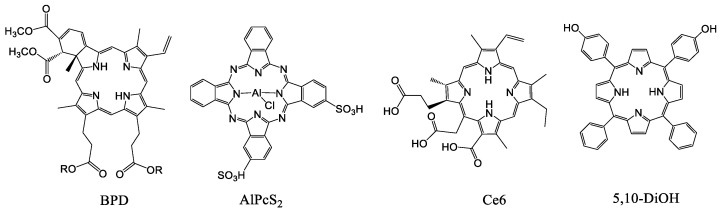
Chemical structures of porphyrins (BPD, AlPcS_2_, Ce6 and 5,10-DiOH).

The similar approach was used in [136], where the embedment of photosensitizers BPD, AlPcS_2_, Ce6 and 5,10-DiOH into the lipid membrane of liposomes as light triggers promoted the release of the calcein upon irradiation (Figure 9). Liposomal formulations with BPD, AlPcS_2_ or Ce6 released calcein from 90% to 100% after 10 min of irradiation. The highest release rate of 82 ± 7.24% after two min of irradiation at a wavelength of 420 nm was observed for liposomes loaded with 5,10-DiOH. The permeabilization of these liposomes as well as those in [135] occurred at a low irradiation dose of 20 mW/cm^2^.

The photosensitizer sinoporphyrin sodium (DVDMS) and PTX were entrapped into the lipid membrane and aqueous core of liposomes prepared from DPPC, 1,2-distearoyl-sn-glycero-phosphoethanolamine-N-[methoxy(polyethyleneglycol)-2000] (DSPE-PEG 2000), CHO and DOPC [137]. It was shown that, under laser irradiation, DVDMS-PTX-loaded liposomes exhibit better antitumor activity against MCF-7 breast cancer compared to DVDMS- or PTX-loaded liposomes. The treatment with DVDMS-PTX-loaded liposomes induced a significant suppression of cancer cell viability and apoptosis in vitro. In vivo studies proved the excellent anticancer activity of DVDMS-PTX-loaded liposomes due to the synergistic effect of PDT.

**Figure 10:**
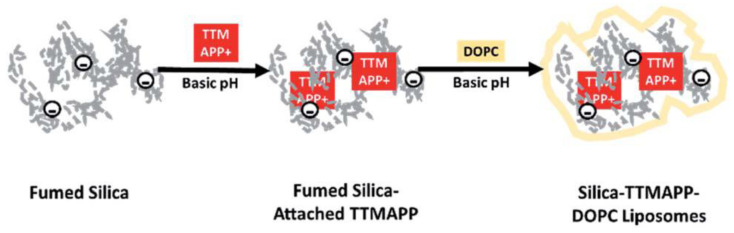
Formation of liposomes loaded with silica-attached TTMAPP porphyrin. Reprinted with permission from [138]. Copyright 2020 Royal Society of Chemistry.

pH-sensitive DOPC liposomes loaded with silica-attached 5,10,15,20-tetrakis(4-trimethylammoniophenyl)porphyrin (TTMAPP) were prepared for targeted delivery of sensitizers to tumor cells (Figure 10) [138]. By controlling the initial pH 7.5 of the liposomes and varying the pH of the aqueous dialysate solution from 2 to 9 by adding acid or alkali, the release of TTMAPP from the lipid membrane was monitored spectrophotometrically after 5 h of dialysis. The release of TTMAPP was about 10% at an alkaline pH (8–9), increasing with increasing acidity of the solution, reaching 80% at pH 2.3. pH-dependent release was also confirmed by singlet oxygen (^1^O_2_) emission upon irradiation of released TTMAPP, as well as fluorescence decays and lifetime images of TTMAPP in the dry lipid film at acidic and alkaline pH. Protonation of silanol groups led to the desorption of TTMAPP from the silica surface and the destruction of liposomes. A toxicity study showed that light-induced apoptosis in DU145, a human prostate cancer cell line, occurred at pH 5.4 and pH 6.3 when treated with silica-TTMAPP-loaded liposomes.

Liposomes embedded with hematoporphyrin monomethyl ether (HMME) as sonosensitizer were prepared to assess the effectiveness of their use for sonodynamic therapy (SDT) in cancer treatment [139]. The HMME-loaded liposomes were 105 nm in diameter with PDI of 0.123 and 33.1 ± 3.56 mV zeta potential. Without sonication these liposomes showed low cytotoxicity; however, after exposure to ultrasound, HMME produced reactive oxygen species (^1^O_2_) which had a significant cytotoxic effect on human MCF-7 breast cancer cells. Liposomes embedded with HMME inhibited tumor growth in vivo more strongly than free HMME after 20 days of treatment.

To enhance delivery efficiency, the synthesized DVDMS-Mn sonosensitizer, a manganese(II) porphyrin complex, was entrapped in DPPC/DSPE-PEG 2000/CHO liposomes [140]. Studies on the U87 human glioma cells showed that the simultaneous treatment with DVDMS-Mn-loaded liposomes and ultrasound resulted in the death of cancer cells. In vivo experiments showed that after intravenous administration of DVDMS-Mn-loaded liposomes, SDT inhibits tumor growth and also significantly increases the survival time of tumor-bearing mice compared to PDT.

In [141], a complex of 5,10,15,20-tetra-p-tolylporphyrin (TTP) with TiO was obtained, followed by loading into liposomes based on lecithin, CHO and 1,2-distearoyl-sn-glycero-3-phosphoethanolamine-N-[folate(PEG)-2000]. The system was expected to have a more efficient photocatalytic effect due to the ability of the TiO-porphyrin complexes to catalyze the conversion of H_2_O_2_, which is abundant in tumors, to singlet oxygen (^1^O_2_) through the formation of a monoperoxo complex between TiO-TTP and H_2_O_2_. It was shown that TiO-TTP loaded liposomes reduced the hypoxic state of the tumor and also inhibited the tumor growth using PDT due to the sufficient amount of oxygen.

The problem of oxygenation was also solved by reducing the consumption of oxygen by cancer cells due to the inclusion of metformin, a hypoglycemic agent, in the liposomal formulation with porphyrin [142]. The hydrophilic metformin and hydrophobic Ce6 (*h*Ce6) modified with hexylamine groups were entrapped into the corresponding aqueous core and lipid membrane of liposomes prepared from DPPC, DSPE-PEG 5000, and CHO. Photoacoustic imaging in vivo and ex vivo immunofluorescence staining showed that tumor oxygenation was greater with intravenous administration of liposomes loaded with metformin and *h*Ce6 in comparison with free drug. In addition, tumor reduction in mice after PDT was significantly higher after intravenous administration of these liposomes than *h*Ce6-loaded liposomes.

In order to reduce phototoxicity, increase the efficiency and selectivity of PDT, a photosensitizer, *h*Ce6, together with NIR dye 1,1′-dioctadecyl-3,3,30, 3′-tetramethylindotricarbocyanine iodide (DiR) were entrapped into the lipid bilayer of DPPC-DSPE-mPEG 5000-CHO liposomes [143]. The fluorescence and PDT effect of *h*Ce6 in DiR-*h*Ce6-loaded liposomes was inhibited by DiR due to fluorescence resonance energy transfer and activated by irradiation with a 785 nm NIR laser. Thus, non-activated DiR-*h*Ce6 liposomes have much lower skin phototoxicity in comparison with the *h*Ce6-loaded liposomes.

In [144] it was proposed to take advantage of the hypoxic state of the tumor for its treatment. Tirapazamine (TPZ) showing strong antitumor cytotoxicity under anoxia, was loaded with Ce6 into the pH-sensitive liposomes of 1,2-dioleoyl-sn-glycero-3-phosphoethanolamine, DSPE-PEG 2000 and CHO. Further, the liposomes were coated with a hybrid membrane based on platelets and red blood cell as an outer shell to prolong blood circulation time. Liposomes had a good ability to accumulate and retain in the tumor due to the biomimetic surface coating. When exposed to ultrasound, Ce6 produced toxic reactive oxygen species for SDT, and the hypoxic microenvironment activated TPZ. The implemented synergistic treatment also significantly inhibits lung metastasis of 4T1 breast cancer cells and B16-F10 melanoma cells.

**Figure 11:**
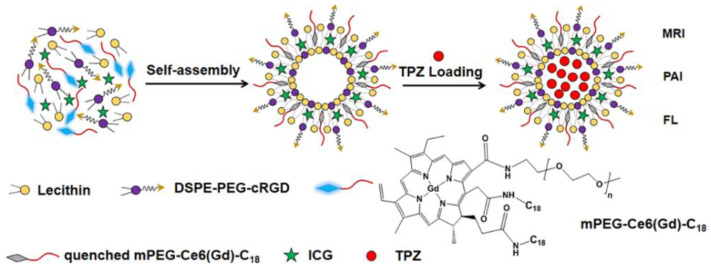
Formation of liposomes loaded with mPEG-Ce6-C_18_. Reprinted with permission from [145]. Copyright 2019 American Chemical Society.

The amphiphilic molecule mPEG-Ce6-C_18_ was entrapped into the lipid bilayer of liposomes prepared from lecithin and DSPE–PEG 2000–thiolated cyclic Arg-Gly-Asp-D-Phe-Lys peptide (cRGD) (Figure 11) [145]. The indocyanine green (ICG) capable of suppressing the PDT activity of Ce6, thereby reducing the side effects on healthy tissues, and TPZ with significant antitumor activity under hypoxic conditions were encapsulated in Ce6 loaded liposomes. The resulting liposomes were further bound with GdCl_3_ as a contrast agent to form ICG/TPZ@Ce6-Gd^III^ liposomes. After targeted delivery to the tumor provided by cRGD, under the action of NIR (808 nm) laser radiation, the liposomes were disrupted by ICG-induced hyperthermia, followed by the release of Ce6 and TPZ. As in [144], a synergistic antitumor effect on A549 lung cancer cells was observed upon sequential activation of Ce6 and TPZ.

**Figure 12:**
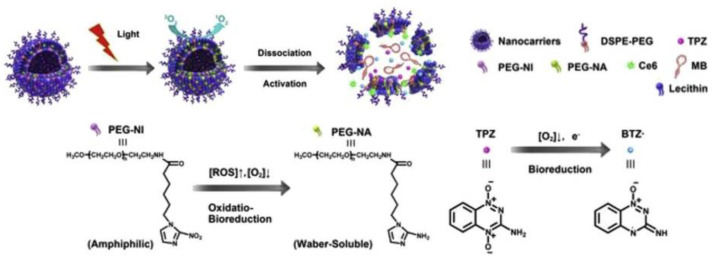
Dissociation of the liposomes with the release and activation of Ce6 and TPZ. Bioreduction of PEG-NI and TPZ. Reprinted with permission from [146]. Copyright 2018 Elsevier.

The hydrophobic Ce6, hydrophilic TPZ and miRNA-155 gene probe were embedded into the lipid bilayer and aqueous core of liposomes based on a mixture of lecithin, CHO, DSPE-PEG 2000 and 2-nitroimidazole derivative linked to PEG (PEG-NI), respectively (Figure 12) [146]. It was assumed that under the laser irradiation and hypoxic conditions, reduced coenzyme II (NADPH) can catalyze the reduction of the –NO_2_ group in the PEG-NI conjugate followed by the formation of the hydrophilic PEG-NA. As a result, liposomes were destroyed with the release and activation of Ce6 and TPZ for synergistic action on cancer cells. The miRNA-155 gene probe showed fluorescence upon interaction with the target, which made it possible to distinguish tumor cells from normal cells during PDT.

The hypoxic tumor microenvironment was also used to selectively destroy the extracellular matrix (ECM) of the tumor in order to improve the effectiveness of cancer treatment [147]. First, olagenase encapsulated in pH-sensitive nanoscale coordination polymers (NCPs) was loaded in liposomes prepared from DPPC, DSPE-PEG5k and CHO. After intravenous administration in tumor-bearing mice, olagenase@NCP-PEG liposomes effectively accumulated in the tumor and were then destroyed by the acidic conditions of the tumor. The released enzyme damaged collagens, the main component of the ECM of the tumor, led to an increase in tumor perfusion and a decrease in hypoxia. Further, liposomes embedded with a complex of modified Ce6 with ^99m^Tc^4+^ were injected intravenously. It was shown that the sequential administration of two types of liposomes leads to the enhanced PDT effect in vivo as compared to the administration of only Ce6-loaded liposomes due to better penetration of latter into the partially destroyed ECM of the tumor. In [148], an increase in therapeutic sensitivity, suppression of tumor growth and metastasis in vivo was also realized due to the destruction of ECM, a decrease in the biomechanical properties of Taxol-resistant tumor cells during PDT using PC/DSPE-PEG/DSPE-PEG-RGD liposomes loaded with porphyrin P-18.

Photosensitizer Ce6-polyvinylpyrrolidone (Photolon) was loaded into liposomes to reduce its phototoxicity upon irradiation [149]. The authors, using their own patented gel hydration technology, obtained unilamellar vesicles with a size of 124.7 ± 0.6 nm, PDI = 0.055 and −5 mV zeta potential with high encapsulation efficiency (93 ± 6%) of Photolon. The liposomal formulation did not cause toxicity in *S. scrofa f. domestica* or dark cytotoxicity in cells in vitro and within 10 min was effectively accumulated by macrophages, the cells involved in origin of atherosclerotic plaque formation, but not by vascular smooth muscle cells or human umbilical vein endothelial cells (HUVECs). When irradiated with a laser, Photolon loaded in liposomes generated reactive oxygen species, which caused a cytotoxic effect in macrophage cell line, but not in accompanying vascular tissue.

**Figure 13:**
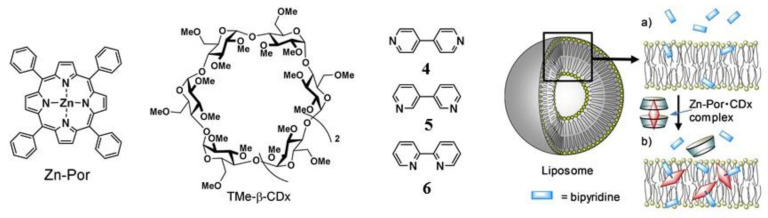
Formation of liposomes loaded with Zn-Por, TMe-β-CD and bipyridines. Retention of bipyridines in the lipid membranes (**a**) before and (**b**) after the addition of the Zn-Por• TMe-β-CDx complex. Reprinted with permission from [150]. Copyright 2019 Wiley Online Library.

The porphyrin complex with zinc (Zn-Por) was added to the liposomal formulation in order to solve the problem of incorporating hydrophilic molecules into the hydrophobic lipid membrane (Figure 13) [150]. 1,2-Dimyristoyl-sn-glycero-3-phosphocholine liposomes were prepared with the addition of hydrophilic 4,4′-bipyridine (4), 3,3′-bipyridine (5), or 2,2′-bipyridine (6). The leakage of 4-6 from liposomes was 93%, 78% and 48%, respectively. When a water-soluble inclusion complex of Zn-Por with TMe-β-CD was added to liposomes, the Zn-Por complex was completely transferred from the TMe-β-CDx cavities to the lipid membrane of the liposomes. Due to the coordination of Zn in the Zn-Por complex with the nitrogen atom of 4 or 5, a significant decrease in leakage to 42% and 23% was observed for hydrophilic 4 and 5 accordingly. The percentage of leakage in the composition with 6 did not change due to steric hindrances preventing the coordination of Zn with the nitrogen atom of 6.

Meso-tetrakis(4-sulfonatophenyl) porphine (TPPS_4_) together with the contrast agent, iodixanol, were entrapped into the aqueous core of positively charged PEGylated liposomes prepared on the basis of DPPC, 1,2-dioleoyl-3-trimethylammonium-propane (DOTAP), 1,2-distearoyl-sn-glycero-3-phosphoethanolamine-N-[aminoPEG 2000] and CHO [151]. In the TPPS_4_/iodixanol-loaded liposome, being in spatial proximity, the heavy atom of iodine affects the pi-electronic system of the photosensitizer, which leads to an increase in the production of singlet oxygen. The study of PDT effectiveness in vitro showed that TPPS_4_/iodixanol-loaded liposome has a higher phototoxicity compared to TPPS_4_-loaded liposomes, mixture of TPPS_4_/iodixanol and free TPPS_4_. Moreover, the intracellular fluorescence was more intense for liposomal formulations than for free TPPS_4_ due to the better internalization of liposomes in cancer cells in vitro.

To solve the problem with the water solubility and photostability, chlorins were loaded in liposomes prepared from POPC and DOTAP [152]. In vitro assessment of photodynamic activity of liposomal formulations against bacteria and fungi showed that chlorins reveal high activity against Gram-positive bacteria *Enterococcus faecalis* and *Staphylococcus aureus*. The decrease in the growth of Gram-negative bacteria *Escherichia coli* occurred to a lesser extent. However, Gram-negative bacteria *Pseudomonas aeruginosa* as well as the fungi *Trichophyton mentagrophytes* and *Candida albicans* did not respond to PDT with chlorin-loaded liposomes. It is well known that Gram-negative bacteria and fungi are often more difficult to treat with photodynamic antimicrobial chemotherapy due to the specific structure of the cell wall of these microbes.

**Figure 14:**
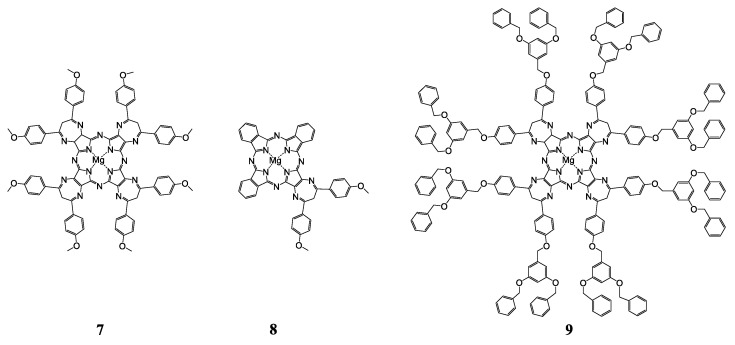
Chemical structures of Mg-porphyrazines.

Hydrophobic Mg-porphyrazines 7–9 (Figure 14) were embedded into the negatively charged l-α-phosphatidyl-DL-glycerol (PG)/POPC liposomes, as well as positively charged DOTAP/POPC liposomes [153]. It was shown that liposomal formulations with 7 and 9 exhibit a slight antitumor activity on the human prostate carcinoma cell line, there was a decrease in cell viability to 65% and 80%, respectively, in comparison with free 7 and 9. Liposomal formulations with sterically unhindered 8, DOTAP/POPC-8 and PG/POPC-8, showed high cytotoxicity upon irradiation with IC_50_ of 0.161 ± 0.002 mM, 0.166 ± 0.058 mM under normoxic conditions and 0.600 ± 0.357 mM, 0.378 mM ± 0.002 mM under hypoxia, respectively. In addition, DOTAP/POPC-8 liposomes exhibited a photodynamic antimicrobial effect against strains of planktonic bacteria (*Staphylococcus aureus* ATCC 25923), in contrast to PG/POPC-8 liposomes.

The talaporfin sodium and anticancer drug, gemcitabine hydrochloride (GEM), were co-encapculated into liposomes based on 1,2-distearoyl-sn-glycero-3-phosphocholine, 1,2-dioleoyl-sn-glycero-3-phosphoethanolamine, CHO and DSPE-PEG 2000 [154]. In vitro studies have shown that in the absence of a radiation source, talaporfin/GEM-loaded liposomes did not show anticancer activity, while with NIR laser irradiation, strong cytotoxicity was found towards the EMT6/P breast cancer cell line, higher than for talaporfin-loaded liposomes and much stronger than for GEM-loaded liposomes. This means that talaporfin acted not only as a trigger for the release of GEM by irradiation, but also as a PDT agent.

In [155], biocompatible hollow nanoparticles of calcium-polydopamine carbonate (CaCO_3_-PDA) were coated with lipid bilayers of sodium 1,2-dioleoyl-sn-glycero-3-phosphate, DPPC and CHO, followed by DSPE-PEG modification. Photosensitizer Ce6 loaded into the obtained liposomes was easily released in a weakly acidic environment due to the high sensitivity of nanoparticles to pH and their rapid degradation under these conditions. The increased Ce6 photoactivity was observed in tumors at low pH due to the increased production of reactive oxygen species. In addition, the established high affinity of PDA for the transition metal ions (Fe^3+^, Zn^2+^, Mn^2+^ and Co^2+^) makes it possible to use liposomes for bioimaging. It has been shown that at normal physiological pH, Ce6@CaCO_3_-PDA-PEG nanoparticles exhibit weak fluorescence due to quenching of the Ce6 signal in the presence of PDA, which effectively reduces skin damage during PDT in vivo.

In summary of this section, the described work on lipid formulations with different types of macrocycles have demonstrated that non-covalent modification of liposomes with CDs or calixarenes significantly increases the stability of systems, and in the case of porphyrins, it solves a number of problems that limit their use as photosensitizers. Since the inclusion of highly hydrophobic molecules in liposomal formulations destroy the lipid membrane, resulting in rapid release of the cargo, the use of the DCL system, where CD acts as a host for the hydrophobic molecule, may solve the problem related to the stability of the liposomes. In particular, a number of studies have shown that when a drug has a higher affinity for CD than for the lipid bilayer, drug release can be delayed. The presence of CD prolongs the shelf life of liposomes in aqueous and lyophilized forms. The modification of liposomes with calixarenes, capable of forming hydrogen bonds with phospholipids or bearing a negative charge on the upper rim, also leads to an increase in the stability of the liposomal system and a decrease in the rate of drug release. A large number of works on non-covalent modification of liposomal formulations with porphyrins is devoted to increasing the efficiency and selectivity of tumor damage in PDT in vivo. The disadvantages of porphyrins used as photosensitizers and sonosensitizers are partially eliminated by their incorporation into liposomes of phospholipids. Lipid formulations with porphyrins or metal–porphyrin complexes exhibit higher toxicity to cancer cells, arrest the tumor growth and/or reduce the tumor, inhibit the metastasis and reduce phototoxicity upon irradiation. Additional surface modification of liposomal formulations with porphyrins can improve functions such as increased affinity for cancer cells, blood circulation time of liposomes, PDT effect of photosensitizer (including antimicrobial effect) and lymphocyte immune response. Thus, the inclusion of macrocycles in liposomes is a promising strategy for improving their functional activity and overcoming their limitations as therapeutic and diagnostic agents.

## 3. Hybrid Nanostructures with Silica-Like Surface

### 3.1. Mesoporous Silica Nanoparticles (MSN) as Perspective Vehicles of New Generation for Drug Delivery

In the recent 20 years, drug delivery systems on the basis of mesoporous nanoparticles of silicon dioxide, carriers of new generation, are intensively designed. They are widely examined as effective and harmless vehicles for many pharmaceutics, genes and visualizers (probes and contrast agents) in the diagnostics and therapy of many diseases [156]. Mesoporous particles have been known since the 1970s [157]. In accordance with classification suggested by M.M. Dubinin in 1972 [158], mesoporous material is a composition with radius of pores in the range of 2–100 nm, which specific surface area *S* reaches 0.5–2 m^2^/g. The first nanocomposite with ordered distribution of pores by size was obtained in 1990–1992 by Japanese and American researchers [159,160]. This product was called Mobil Crystalline Materials or Mobil Composition of Matter (MCM-41) and became the ancestor of the series of M41S mesoporous silica with P6mm space-group symmetry [161]. These reports formed the basis for the series of investigations started from 1995 and dedicated to the design of novel types of mesoporous silica materials with variable composition of reactants and reaction conditions. Depending on the synthetic approach their physicochemical properties and characteristics of porosity could significantly differ between each other. For example, SBA-type (Santa Barbara Amorphous) mesoporous silica particles have a 2D hexagonal structure similar to MCM-41, but with the larger pores and the thicker internal walls [162]; hexagonal mesoporous silica particles have disordered hexagonal structure and walls of high thickness [163]; KCC-1 particles are characterized by a three-dimensional dendritic and fibrous structure composed of disordered network of pores [164]. Folded sheets mesoporous materials (FSM-16) are composed of sheets of mesoporous material and have high thermal and hydrothermal stability [165]. MSU-type particles (Michigan State University) have a disordered structure of pore channels and small size of particles with significant patterns of mesoporosity [166]. Among other types of mesoporous silica particles, FDU-type (Fudan University Material) [167] and KIT-type (Korean Technological University) particles [168] were described. Some selected types of mesoporous silica nanoparticles and their physicochemical characteristics are listed in Table 2 [169].

Pores in mesoporous silica particles could be structurally arranged in various ways and, therefore, three different basic types of silica compositions could be established: (a) MCM-41 (hexagonal arrangement of pores; (b) MCM-48 (cubic arrangement of pores [170]); and (c) MCM-50 (lamellar arrangement of pores [170]).

There are several ways of preparation of mesoporous materials: methods of soft and hard templates, the Stober method, approaches employing colloid templates, aerogel technique [171] and spray pyrolysis method [172]. It should be noted that the latter method can be used to obtain mesoporous particles on an industrial scale, since all stages of the synthesis are carried out in one container. In the framework of this review only two of them will be considered: the soft template method (interfacial synthesis) and hard template method (core–shell technique). In the first technique organic template molecules (vesicles, emulsions (oil-in-water and air-in-water), polymeric micelles) are used to fabricate the structure of future voids with their further removing by physical or chemical ways [173]. The main disadvantage of this method is the lack of possibility to control the size and the thickness of particle shells, since obtained dispersions are unstable. Hard template method includes the precipitation of silicon dioxide on the template prepared from other materials (for example, polystyrene [174] or inorganic spheres, multiwalled carbon nanotubes [172] with its subsequent removing by physical or chemical methods, in particular, by dissolution or calcination. Compared to the previous technique, with the hard template method the shape and the size of voids of mesoporous silica nanoparticles could be easily and precisely regulated by the selection of certain preliminarily prepared assemblies [175].

**Figure 15:**
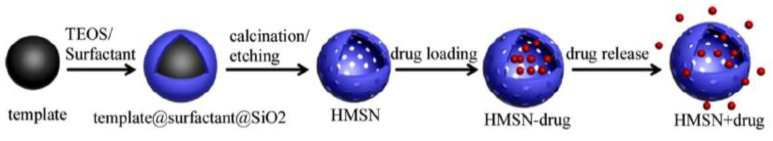
Schematic illustration of preparation of hollow mesoporous silica nanoparticles (HMSN) and drug loading-release. Reprinted with permission from [188]. Copyright 2017 Royal Society of Chemistry.

Unique physicochemical properties are responsible for the practical application of mesoporous silica nanoparticles in various technologies including biomedical and pharmaceutical spheres. In particular, since 2001 they are considered as nanocontainers for drug delivery [169]. United States Food and Drug Administration (USFDA) classifies mesoporous silica particles as agents belonging to the Generally Recognized as Safe category, i.e., they are recognized as one of the most suitable nanoplatforms for clinical applications [176]. For example, recently USFDA has approved the first diagnostic system based on of mesoporous silica particles called Cornell dots for the first phase of clinical trials [177]. Mainly, these results are due to advantages of mesoporous silica structures over other drug delivery systems. In particular, inorganic origin of MSN walls prevents mechanical, thermal and biological degradation of encapsulated cargo, which provides safe and effective drug delivery [178]. High specific *S* (700–1200 m^2^/g) and variable pore size (2–50 nm) are key factors determining type and amount of drug that could be encapsulated into MSN [179]. For instance, downsizing of pores results in the decrease of the drug loading, and the rate of cargo release is reduced due to the higher density of molecules packed in mesopores. Increase in *S* of pores improves the loading of drugs and accelerates the rate of their release. Moreover, mesoporous silicas can have sizes of particles in the desired range, which influences several parameters of drug carriers, i.e., the time of semi-ejection, extravasation across the vascular tree and absorption by macrophages [180,181]. Optimal MSN diameter favoring the accumulation of vehicle in target tissue and providing more prolonged period of semi-ejection is in the range of 50–250 nm; therefore, in the majority of cases mesoporous silica nanoparticles meet the criteria for biomedical application. [182]. Another advantage of MSNs is their capability to biodegrade [183] and excretion from the organism [184,185]. All the mentioned features of mesoporous silica nanoparticles strongly encourage intensive research and design of these systems as delivery vehicles for drugs of wide spectrum of activity. For example, in [186] mesoporous silica particles of variable size (the range of diameter was 90–300 nm) and pores (3.5 nm to 7 nm) were tested as carriers for drug resveratrol. The formulated drug demonstrated higher anti-inflammatory and anticancer activity toward human colon carcinoma cell Caco-2 in comparison with resveratrol solution and suspension. In [187] potential applicability of MSNs of MCM-41 and SBA-15 type as drug delivery systems for clofazimine, an antibiotic of wide spectrum, was demonstrated. The authors of [188,189] successfully used hollow mesoporous nanospheres (Figure 15) with diameters around 400 nm for encapsulation of ibuprofen. Obtained systems were characterized by prolonged drug release up to 45 h [188] and self-activating luminescence properties [189].

In [190] a novel MSN-based drug delivery system with a potential in the treatment of lymphoma was suggested. It was loaded with isoimperatorin and exhibited high capability to induce apoptosis of OCI-LY10 tumor cell line with no cytotoxic effect toward normal organs of mice. In [191,192] nanoporous silica dioxide of MCM-48 and SBA-15 types were used as vehicles for delivery of anti-inflammatory drugs INM and naproxen. These nanoformulations were characterized by variable degree of encapsulated drug release depending on pH of media in the range of 50–100% [191] and 30–80% [192]. Ibuprofen was successfully encapsulated into MCM 41 mesoporous silica nanoparticles in [193]. This system was characterized by 30% *w*/*w* loading of drug into carrier. The authors of [194] used MCM-41 type MSNs for transdermal delivery of methotrexate. The obtained drug delivery system exhibited bioavailability toward HaCaT keratinocytes and a higher accumulation of formulated drug in comparison with free methotrexate. Report [195] was dedicated to the encapsulation of peptide bactofencin A in SBA-15 type mesoporous silica nanoparticles. Encapsulated drug demonstrated superior antibacterial properties in comparison with free drug and was capable to suppress the growth of *Staphylococcus aureus* bacteria by ~80%.

### 3.2. Modified Mesoporous Silica Nanoparticles as Drug Delivery Systems

One of the key structural features determining biological activity of mesoporous silica nanoparticles is the presence of siloxane (Si-O-Si) and silanol groups (Si-OH) on their surface. In particular, cytotoxicity of MSNs depends on the number of silanol groups, since the proton in a silanol group could interact with various endogenous targets: (1) with membranes of erythrocytes, which leads to hemolysis [196]; (2) with phospholipids based on tetraalkylammonium resulting in the fabrication of surface silanolate and cytolysis; (3) with cell membranes, inducing fabrication of reactive oxygen species and cell death through necrosis or apoptosis [177,197]. At the same time, the number of silanol groups is related to the porosity of MSN and its size. The presence of pore voids on the surface reduces the interaction between silanol groups and cell membrane, which prevents death of the cell [196,198]. Moreover, the presence of the silanol groups on the surface of nanoparticles of silicon dioxide facilitates their functionalization. There are three areas for functionalization: overall framework, pores and external surface of silica particles [199]. Rich possibilities and various ways of modification of both interior and superficial MSN surfaces produce a wide variety of mesoporous materials based on silica as impending candidates for biomedicine and drug delivery. By selecting functional groups on the mesoporous carrier (Table 3 [174]) and taking into account the chemical nature of the molecule of the encapsulated drug, it is possible to influence its loading and change its release profile. It is also possible to significantly increase the loading of encapsulated substance (DOX, ibuprofen) by changing only the pore volume (PV) and specific *S* of the mesoporous carrier (in the case of HMSNs PV = 1.04 cm^3^/g and surface area *S* = 1210 m^2^/g, while PV = 0.73 cm^3^/g and *S* = 1106 m^2^/g for MCM-41).

The choice of modifying groups can significantly affect the functional the properties of MSNs as drug delivery systems. For instance, functionalization of MSNs by targeting ligands (sugar fragments, peptides, antibodies [200], nucleic acids, various low molecular weight compounds [201], etc.) that could recognize and bind to cellular receptors improve their targeting properties and therapeutic efficiency. There are two common ways to modify mesoporous silica: co-condensation and postsynthetic functionalization [156]. In the case of co-condensation, the internal surface of pores is functionalized, while in the case of postsynthetic approach mostly external surface could be modified [197,202]. Co-condensation (also called direct synthesis) includes the stage of insertion of organic species into the framework of silica dioxide during synthesis process using the sol-gel method [203]. Postsynthetic functionalization represents the modification of MSN surface by organic species after synthesis of the nanoparticles [204].

Since there are a lot of various approaches of the modification of MSNs, only selected types of modification will be considered in detail. In particular, MSNs modified by synthetic and natural polymers, as well as mesoporous silica nanoparticles decorated by lipids will be discussed.

### 3.3. Mesoporous Silica Nanoparticles Modified by Synthetic Polymers

**Figure 16:**
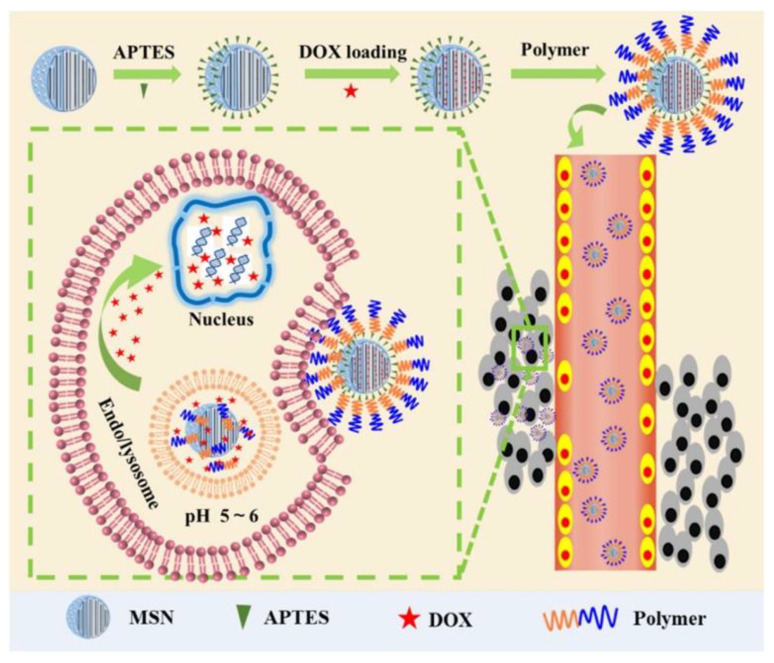
Schematic illustration of the preparation of Polymer@MSN-DOX and pH-triggered release of DOX. Reprinted with permission from [210]. Copyright 2019 Elsevier.

The first big group of modified mesoporous silica nanoparticles is their conjugates with synthetic polymers. In the majority of cases, pH-responsive polymers are picked, which have capabilities to change configuration upon slight changes of pH, that in turn could provide controlled release of encapsulated cargo. Therefore, these systems are often used as drug delivery systems [205,206,207]. For example, poly-(2-vinylpyrrolidone) was involved in [208] for modification of the surface of mesoporous silica, where the composition was used as an agent for delivery of antitumor drug 5-fluorouracil. Application of this composition allows one to reach pH-dependent prolonged release of encapsulated drug (about 82% of released cargo from the total amount after 70 h). Polyacrylic acid was also used for covalent modification of the surface of mesoporous silica particles for peroral delivery of DOX [209]. Obtained DOX delivery system made it possible to interrupt premature release of encapsulated drug before reaching the colon, as was desired. Another example of delivery system for DOX is MSN modified by amphiphilic block copolymer poly(poly(ethylene glycol) methylether methacrylate-*co*-p-(2-methacryloxyethoxy)benzaldehyde) [210]. The system formed is capable to release content at pH 5.0 (64% of released drug from the total amount after 72 h) and effectively penetrate into HepG2 cells (Figure 16).

The authors of [211] decorated the surface of mesoporous silica by glucose oxidase and polyelectrolytes (sodium polystyrene sulfonate and poly(allylamine hydrochloride)), which makes it possible to charge particles positively and target them towards cancer cells. Grafted enzyme stimulates oxidative processes in the cancer cell, which results in pH decrease. This process helps destabilize the polyelectrolytes, which increases the rate of release of encapsulated cargo. Copolymer poly(ethylene glycol)methyl acrylate-*co*-itaconic acid was used for functionalization of the surface of mesoporous carrier containing a luminescent dye 10-phenylphenothiazine and cisplatin [212]. This nanoformulation was characterized by improved stability in water, high encapsulation efficiency of cisplatin and capability of pH-controlled cargo release. Gold nanoparticles (AuNP) were inserted into polydopamine (PDA) for modification of the surface of mesoporous silica in [213], and the fabricated composition was examined as DOX delivery system. PDA contains catechin functional groups that provide pH-sensitivity of the obtained formulation in aqueous media. MSN@DOX-PDA-AuNPs nanostructures exhibited synergetic photo- and chemotherapeutic effect with high rate of DOX release in acidic media under near infrared laser irradiation (19% of cargo released during 15 h). Hyperbranched polymer with terminal amino groups combined with folic acid were used for modification of the surface of mesoporous nanoparticles in [214]. The obtained system had high biocompatibility and increased capability to be absorbed by lysosomes of HeLa and A549 cancer cells. The presence of hyperbranched polymer made it possible to increase loading of tetrahexin up to 27% and induce pH-sensitive release of encapsulated cargo (drug release could be prolonged up to 20 h at pH 7.4).

Alongside pH-sensitive polymers, their thermosensitive counterparts are also used for modification of mesoporous silica nanoparticles. Thermosensitive polymers are characterized by capability to undergo a sharp phase transition from globular structure to less ordered structures at a certain temperature [215,216], which provide selective and controllable release of encapsulated cargo. In particular, the authors of [217] coated MSN with copolymer poly(N-isopropylacrylamide-*co*-methacrylic acid) and used the obtained composition for DOX delivery. The particles were able to release encapsulated drug in conditions of pH variation and heating up to 37 °C under an alternating magnetic field. This resulted in a higher efficiency of examined therapeutic composition in comparison with the free drug. In [218] poly-N-isopropylacrylamide (PNIPAM) was grafted on the internal and external surface of silica nanoparticles modified with organoalkoxysilane bearing RAFT agent, with the fabricated formulation evaluated as a DOX delivery system. Thoughtful selection of the hydrocarbon fragment in RAFT allowed one to change the location of functional groups and control the conjugation of the polymer with silica matrix. External grafting of polymer in the case of PNIPAM–COOH–MSN system was presented as a thermosensitive system with superior encapsulation efficiency (61%) and drug release properties (54% of released drug from the total amount during 24 h at 20 °C). Aliphatic polyurethaneamine (PUA) was used in [219] for decoration of MSNs as a smart polymer sensitive to temperature and pH; the obtained nanovehicle was used for DOX delivery, where PUA functioned as a reversible valve that allowed the authors to regulate the rate of drug release under influence of the temperature due to stretching and compression of its polymer chains.

Special place is occupied by MSNs modified by polymers responsive to electric field. For example, in [220] poly(3,4-ethylenedioxythiophen) doped by poly[4-styrenesulfonic acid)-co-maleic acid] and modified by bipyridinium was used for MSN functionalization. The prepared vehicle was examined as a carrier of fluorescent dye Rhodamine B. Pores of obtained modified mesoporous particles were closed upon addition of negatively charged polysaccharide heparin due to its interaction with bipyridinium. An external electric field results in reducing of the charge of bipyridinium with following detachment of heparin and opening of pores. Electromotive force about 600 mV was demonstrated as sufficient for the release of encapsulated Rhodamine B, which was absorbed by HeLa cells through phagocytosis mechanism.

Another approach to enhance the specificity of MSNs toward certain cells, especially cancerous, is their conjugation with long-chained polymers (stealth-polymers) combined with ligands or antibodies. Stealth-polymers provide dispersability and biomembrane permeability of the polymer–silica conjugate, inhibit its absorption by reticuloendothelial system and prolong circulation of the carrier in vivo [221,222]. PEG and polypropyleneglycol (PPG) are often applied as stealth-polymers. For example, a combination of PEG or PPG with lactose as a targeting ligand was used for decoration of the surface of mesoporous silica particles, and the prepared system was tested as a vehicle for DOX delivery [173]. The conjugate with PPG exhibited high cytotoxicity toward HepG2 cells (IC_50_ = 0.07 mg/mL). PEG with average molecular weight about 6000 g/mol combined with folic acid and rosin as an organic additive was applied for modification of the surface of mesoporous silica [223]. The choice of this ligand was caused by the fact that folic acid receptors are overexpressed on the surface of the majority of human cancer cells like ovary, breast, colon, lung and prostate ones. Obtained formulation was tested as DOX delivery systems. The designed DOX@MSN-x-PEG-FA system exhibited pH-dependent release of drug and caused apoptosis in significant amounts. In [224] for delivery of 5-fluorouracil, the authors designed mesoporous silica nanoparticles decorated by poly(oligo(ethylene glycol) monomethyl ether methacrylate) functionalized by cRGD-peptide into HCT116 colon cancer cell lines. The obtained formulation demonstrated improved internalization ability and increased accumulation capacity in these cancer cells both in vitro and in vivo. The authors of [225] immobilized β-CD in combination with azobenzene/galactose-grafted polymer (GAP) on the surface of mesoporous silica nanoparticles. The latter is a well-known ligand for targeting asialoglycoprotein receptors of HepG2 cells and controllable release of encapsulated cargo. Fabricated conjugate was tested in terms of the applicability as a DOX delivery system. The formed MSN@β-CD@GAP@DOX system was characterized by increased antitumor activity, which could be induced by two factors: UV irradiation or the change of pH. In [226] for DOX delivery MSNs were modified by two polymeric layers consisting of polyethyleneimine (PEI) and hyaluronic acid that is a ligand targeted to CD44 receptors overexpressed in lung, breast, pancreas and kidney cancer cells. HA-PEI-MSN-DOX particles demonstrated a capability to actively target A549 cancer cells and an ability to avoid endocytic decomposition pathway, which increase therapeutic efficiency. This nanoformulation exhibited high cytotoxicity toward A549 cells at low DOX concentrations (only 20% of viable cells for 400 nM of the drug), which is comparable with cytotoxicity of free DOX.

Polymers are often conjugated to the MSN in order to induce certain structural or morphological characteristics (particles size, pore structure, hydrophobic or hydrophilic properties, colloid stability, biocompatibility etc.) that facilitate their application in industry and medicine. For example, in [227] MSNs were modified by poly(amido)amine dendrimers (PAMAM) of the first and second generations for imparting mucoadhesive properties to the carrier. Bioadhesion of these polymers was caused by electrostatic interactions between positively charged dendrimers and negatively charged mucin of urothelium. The authors showed that mucoadhesive properties and release of encapsulated DOX could be controlled by the selection of the number of PAMAM layers immobilized on the surface of MSNs. The designed formulation provided prolonged release of DOX at low pH values. In [228] poly(2-ethyl-2-oxazoline) was used for decoration of the surface of MSNs in order to increase hydrophilicity and biocompatibility of the mesoporous carrier. This polymer is characterized by high stability, low viscosity and absence of toxic products formed during the synthesis. Examination of fabricated formulations in terms of the capability to encapsulate PhE-OH luminescent dye showed that formed composites exhibited an intensive yellow fluorescence upon photoexcitation, which could be used in a variety of biological experiments for visualization purposes. The authors of [229] decorated the surface of mesoporous silica by PEG and PEI containing carbon dots for inducing improved permeability as well as a capability to monitor the distribution and transport of nanoparticles in vivo in real-time mode. This formulation was characterized by high degree of transepithelial absorption and peroral bioavailability. In [230] colloid stability and protection through the fabrication of hydrophilic layer of MSNs were provided by conjugation of silica matrix with copolymer containing PEG and chitosan units. This nanovehicle was documented as safe and effective for in vivo delivery and was characterized by improved 5-fluorouracil loading (up to 65%) and prolonged release of the drug (up to 72 h). The main idea of [231] was to induce colloid stability and sustainability toward various external pH values; therefore, MSNs were decorated by poly(trimethylolpropanetrimethacrylate). This formulation was tested as diclofenac delivery system and is characterized by micrometer size and specific *S* ~500 m^2^/g and high drug loading capability (90%). Application of this carrier allows authors to avoid desorption of diclofenac in acidic media (less than 6% of desorbed diclofenac after 2 h of contact), and therefore it looks attractive for the purposes of peroral delivery. Report [232] was dedicated to enhancement of MSNs lubrication properties upon drug injection into injured arthrodial cartilage. The authors grafted 3-[dimethyl-[2-(2-methylprop-2-enoyloxy) ethyl] azaniumyl] propane-1-sulfonate on the surface of MSN. This polymer forms tenacious hydration layer that remains almost rigid under pressure and responds in a fluidlike manner under shear, resulting in a great reduction in interfacial friction. Application of this composition allowed them to reduce friction by 80% and inhibit release rate of encapsulated test cargo Rhodamine B by almost two times in comparison with unmodified MSNs.

### 3.4. Mesoporous Silica Nanoparticles Modified by Natural Polymers

The other approach to modification of nanosystems on the basis of mesoporous silica is their conjugation with polymers of natural origin. The main idea of this approach is (1) the enhancement of the biocompatibility of the system due to the decoration of the surface of silica particles by endogenous or nontoxic high-molecular weight compounds like proteins, nucleic acids etc.; (2) infusion of the affinity of the system toward various target tissues and cells due to molecular recognition of conjugated polymers and biocomponents. These advantages of mesoporous silica nanoparticles look promising for targeted drug delivery, and below, several chosen examples are considered in terms of their present or future application in biomedicine.

In particular, decoration of the surface of mesoporous nanoparticles by poly-l-lactic acid and chitosan allows authors to construct a pH-sensitive vehicle for anti-inflammatory drug dexamethasone delivery applicable for bone tissue engineering [233] and nanocarrier of thymoquinone for the delivery to glioma cancer cells [234].

**Figure 17:**
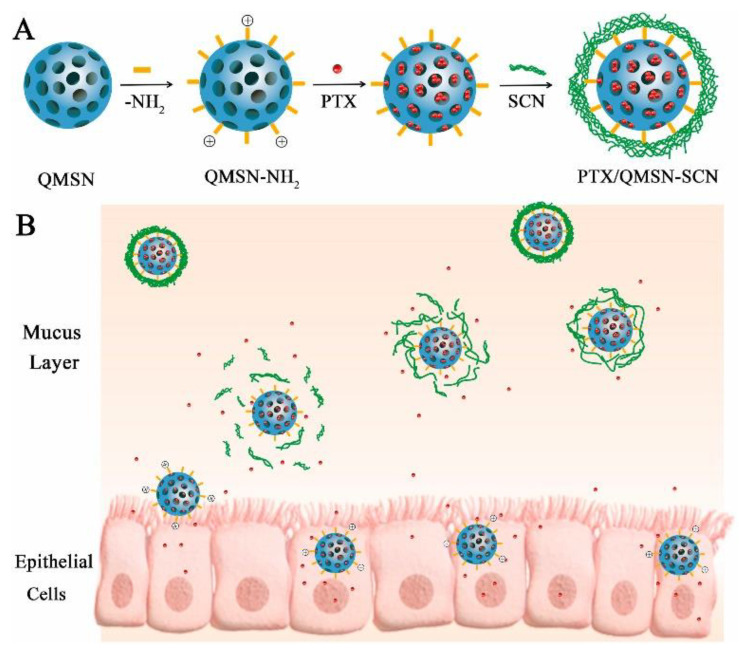
Scheme of protein-decorated mesoporous silica nanoparticles preparation (**A**) and its intestinal uptake (**B**) (here QMSN = CdSe/ZnS quantum dots doped mesoporous silica nanoparticles; SCN = succinylated casein). Reprinted with permission from [235]. Copyright 2020 Elsevier.

However, the most frequently reported type of biopolymers used for modification of mesoporous silica platform are proteins. There are a lot of examples of successful conjugation of silica matrix with polypeptides of various types suitable for several biomedical applications Mainly, it is drug delivery, since these hybrid formulations demonstrate an ability to effectively encapsulate pharmaceuticals, specificity toward certain cell lines, prolonged release profiles and capability to penetrate through various biological barriers. For example, a casein derivative was successfully applied for the preparation of silica/polymer drug vehicle containing encapsulated PTX with increased permeability through gastrointestinal barriers (Figure 17, [235]).

Another protein, bovine serum albumin was immobilized on mesoporous silica nanoparticles that were engineered as core–shell carriers for biologically active compounds. This composition was recommended for various applications such as regeneration of bone tissue [236]. Conjugation of silica particles with peptides is an excellent approach to improve specificity of nanovehicle toward certain cells. For instance, coating of silica particles with Cyc6, a bladder cancer cell specific peptide, made it possible to reach enhanced binding efficiency and specificity of modified nanoparticles for cancer cells of bladder in vitro and in vivo [237]. Another effective example in this direction is immobilization of tumor-homing peptide CREKA on the surface of mesoporous silica nanoparticles for fabrication of the nanocarrier for methotrexate delivery. These formulations were able to prolong the encapsulated drug release up to 60 h and the treatment of cell cultures by obtained formulations showed good viability of MRC-5 fibroblast cells and suppression of HeLa cells [238]. The authors of [239] designed gelatin-coated mesoporous hollow silica nanospheres and used them for glimepiride encapsulation. Formulations were characterized by high drug loading (40%) and more than a 2-fold higher duration of hypoglycemic effect in comparison with commercially available formulations, which allows researchers to recommend them as perspective candidates for the treatment of type 2 diabetes. Mesoporous silica particles decorated by sericin and transferrin were reported as effective carriers of DOX, with high cargo encapsulation parameters, as well as pH-triggered and prolonged release [240,241]. One of the main features of compositions based on proteins or peptides immobilized on the silica matrix is their capability to pass through various biological barriers. In particular, modification of the surface of silica particles by lactoferrin was documented as a successful approach allowing to guide carriers through BBB [242]. Application of polypeptides for decoration of mesoporous silica particles opens opportunities for other potential directions of biomedicine: *ε*-polylysine was documented as a component for fabrication of nanomotor-based removers of Pb^2+^ from the blood, which makes it a perspective candidate for treatment of heavy metal poisoning [243].

### 3.5. Mesoporous Silica Nanoparticles Modified by Lipid Shell

Among approaches to modification of mesoporous silica nanoparticles, special attention is paid to the decoration of the carrier surface by a lipid bilayer. These structures of core–shell type allow authors to combine advantages of lipid bilayer based nanovehicles, i.e., liposomes, (biocompatibility, prolongation of drug release, protection of cargo from premature enzymatic decomposition) [2,244,245,246] and porous silica material (nontoxicity and high encapsulation capability of various cargos). Usually as a material for lipid bilayer fabrication various two-tailed amphiphilic compounds are applied, including 1,2-dioleoyl-sn-glycero-3-phosphoethanolamine (DOPE); 1,2-distearoyl-sn-glycero-3-phosphoethanolamine (DSPE); 1,2-distearoyl-sn-glycero-3-phosphocholine (DSPC), 1,2-dioleoyl-sn-glycero-3-phosphatidylcholine (DOPC), DPPC and their mixtures in various proportions.

Lipid bilayer-coated mesoporous silica particles, also called silicasomes, show a number of useful properties suitable for biomedical application. For example, there is a series of reports dedicated to the development of drug delivery systems based on irinotecan and AZD1080, inhibitor of enzymes responsible for phosphorylation, for treatment of alimentary canal cancers [247,248,249,250,251]. In particular, application of silicasomes was shown to demonstrate improved biodistribution and delivery of AZD1080 to the sites of colorectal tumor CT26 and pancreas KPC cancer models [247]. Similar modification allows authors to achieve excellent encapsulation capability of irinotecan, improved pharmacokinetics and 6-fold higher amount of drug in colorectal and pancreatic cancer cells after 48 h of treatment over free drug and its liposomal formulation [248]. Moreover, lipid-decorated silica particles have perspectives in various approaches of cancer treatment including chemotherapy and chemo-immunotherapy of pancreatic cancer [249,250].

**Figure 18:**
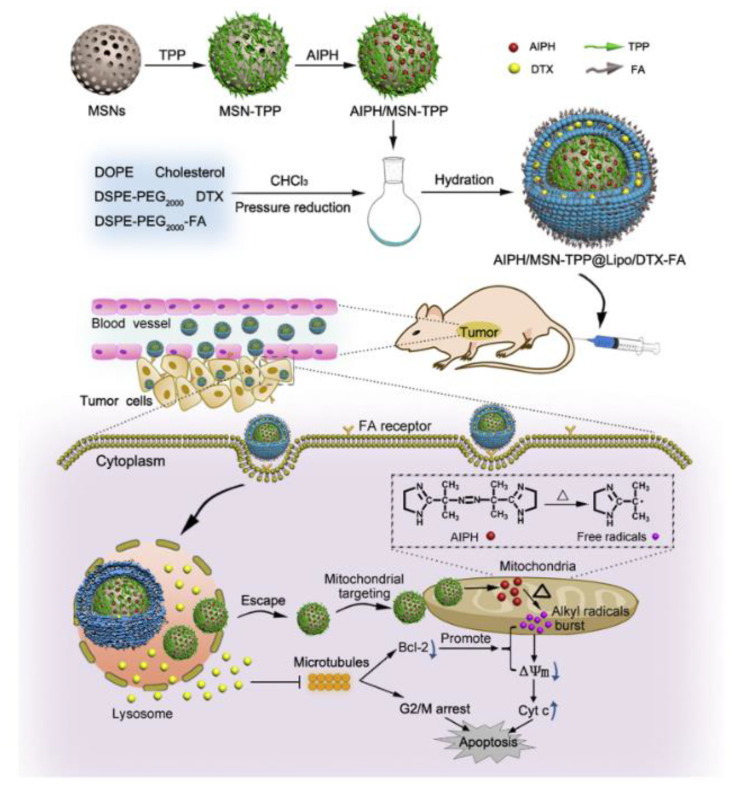
Schematic representation of preparation of MSN particle in lipid shell and mechanism of its antitumor activity (MSN = mesoporous silica nanoparticle; TPP = triphenylphosphine; AIPH = 2,2′-azobis[2-(2-imidazolin-2-yl)propane] dihydrochloride; DTX = drug docetaxel; FA = folic acid; DOPE = dioleoylphosphoethanolamine; DSPE-PEG_2000_ = 1,2-distearoyl-sn-glycero-3-phosphoethanolamine-N-[amino(polyethylene glycol)-2000] (ammonium salt). Reprinted with permission from [254]. Copyright 2020 Elsevier.

All reports concerning lipid-bilayer decorated MSN particles could be divided in several sections using the material of the shell as a determining criterion:

(1) DOPE-based lipid bilayer shells. Decoration of the surface of mesoporous silica particles by lipid shell composed of this lipid was shown to 2-fold increase cellular uptake of zoledronic acid by MCF-7 cancer cells by 2-fold [252] and to act as microenvironment responsive gentamicin delivery agents against *Staphylococcus aureus* bacteria with no toxicity to human organs [253]. Besides this, additional modification of MSN of this type by TPP cation was demonstrated as a successful example of the design of docetaxel delivery system with pH-sensitive release and organelle-targeting properties [254] (Figure 18).

(2) DSPE-based lipid shells. Notably, in the majority of cases this lipid is conjugated with PEG with average molecular weight about 2000 g/mol. The reason of application of this lipid–polymer conjugate is the inducing “stealth” properties to the system, i.e., inability to be spotted by immune system [255]. Transition from the lipid bilayer composed of unsaturated lipid (DOPE) to its saturated counterpart (DSPE) allows researchers to construct drug vehicles for arsenic trioxide aimed on the delivery to MCF-7 and HepG2 cancer cell lines capable to prolong drug release by more than 1.5 times [256] and to increase the targeting efficiency toward glioma cells by 4-fold [257]. Moreover, this lipid shell was used for fabrication of MSN-based drug delivery system of DOX for chemo-photothermal therapy of cancer on the example of MCF-7 cells [258] and nanovehicles for successful co-delivery of docetaxel and DOX demonstrating increased cytotoxicity towards MDA-MB-231 and MCF-7 cancer cell lines [259]. Combination of DOPE and DSPE in the lipid shell-coated MSN was used for preparation of a nanovehicle for the delivery of antibacterial peptide colistin for effective and selective treatment of lung infection *Pseudomonas aeruginosa* with no cytotoxicity toward normal lung epithelial cells [260].

(3) Other lipids-based shells. The shell constructed from the mixture of DPPC and DSPC was applied for the fabrication of nanocarriers of DOX for delivery into U-251 glioblastoma cells, which was characterized by 90% encapsulation efficiency and thermo-responsive release [261]. The other example of coating of this type is soybean PC, which is being combined with an additional layer of calcium phosphate on the surface of MSN, allowing to construct the system for co-delivery of zinc (II) phthalocyanine and DOX. This formulation had pH-triggered controllable drug release properties and induce HeLa cell apoptosis [262]. 1-Palmitoyl-2-oleoylphosphatidylcholine-coated MSNs were described as promising drug vehicles possessing acid-triggered release of encapsulated cargo [263].

At this point, the concept of MSNs is highly developed and the proposed drug delivery systems often exhibit multiple aspects of activity, such as targeting, stimuli-responsive, stealth properties. This allows for a very diverse range of their application. While being relatively slow nanovehicles in terms of cargo release, silica nanoparticles are superior to liposomes in the amount of drug they can hold, but at the same time, can be functionalized by a wide variety of intelligent homing ligands as the liposomes. While non-toxic and non-immunogenic, silica nanoparticles can exhibit some degree of toxicity due to traces of synthetic precursors and catalysts, such as ionic surfactants, acids and alkali. A high density of silanol groups on the surface can be a hazard in a bioenvironment leading to hemolysis or protein denaturation. Yet, a large number of successful examples of developed drug delivery systems based on MSNs, which have outstanding biomedical parameters, allows researchers to expect their rapid advancement to clinical application, as long as the abovementioned limitations are addressed with caution.

### 3.6. Cerasomes

Cerasomes are a hybrid class of organic-inorganic vesicles that have a bilayer membrane and a siloxane surface [264]. Liposome-like morphology allows them to encapsulate hydrophilic and hydrophobic substrates, and the siloxane coat provides an extraordinary morphological stability and durability. Cerasomes consist of cerasome-forming lipids (CFLs) that have three distinguishable parts: the hydrophobic domain, the so-called hydrogen belt, and a headgroup bearing at least one triethoxysilyl moiety [265]. The CFLs are presented in a variety of forms that will be discussed later. An important notion to the CFL structure is that inherently, the triethoxysilyl headgroup is hydrophobic and is therefore disadvantageous for the bilayer self-assembly in water. A precisely controlled preliminary hydrolysis is necessary to enable proper aggregates to form. Originally, Katagiri et al. proposed a CFL that contains an ammonium fragment in the headgroup area, which allows for the lipid to form bilayers without preliminary hydrolysis [266].

**Figure 19:**
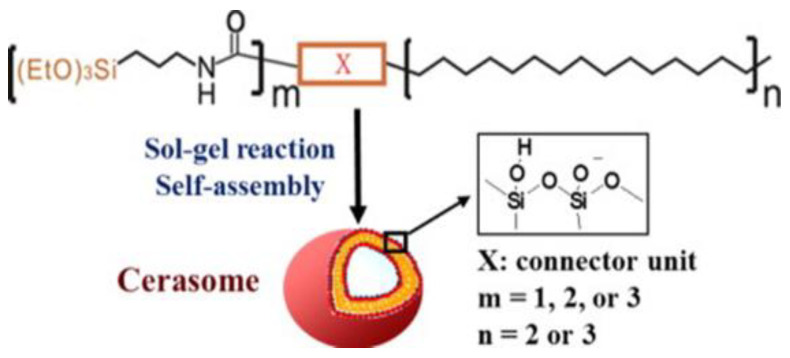
General concept of cerasome: a vesicle that is constructed of cerasome forming lipids that undergo hydrolysis upon forming a bilayer and covalently link with each other via Si-O-Si bonds. Reprinted with permission from [267]. Copyright 2014 Elsevier.

Consequently, the concept of a surface polymerizing liposomal structure has attracted a lot of research interest (Figure 19) [267], so over the last two decades, many contributions have been made to the cerasome concept. Originally, the high degree of siloxane formation was not achievable with lipids to full extent due to the fact that the lipid headgroup cross-section is smaller than that of the hydrophobic domain and full linking is inappropriate in terms of packing parameters for bilayer formation [264]. This could be avoided in two ways: addition of tetraethyl orthosilicate or (3-aminopropyl)triethoxysilane (APTES) or dodecyltriethoxysilane as was proposed by the original authors [268,269]; or by changing the lipid structure, as Liang et al. have established, a structure–property relationship involving the amounts of triethoxysilyl groups and hydrophobic tails in one CFL molecule, and as a result, the hydrophilic as well as hydrophobic substrate release rate can be tweaked by varying the building blocks [270]. It is also expected that overall structural sturdiness can be higher, since 2- or 3-headed CFLs form more siloxane bonds with neighboring molecules [270].

As a novel type of a nanocontainer, cerasomes were immediately subject to a toxicity and pharmacokinetics study, which showed that recoverable acute toxicity is observed at a relatively high concentration of 50 mg/kg of CFL, while cerasomes also provided a significantly prolonged cargo release profile in vivo. The circulation time was higher than that of the conventional liposomes, but lower than typical stealth liposome time, and the sustained drug release resulted in slow cargo release, which was most significant for cerasomes, compared to other liposomes and hybrid nanocarriers in the study. Overall, the morphological stability of cerasomes seems to be the driving factor of their prolonged circulation time in vivo [271]. The structural stability of cerasomes is also advantageous for production of smaller sized vesicles, since covalently bound CFLs are not so prone to fuse with neighboring membranes, as are conventional phospholipids due to curvature induced strain on the membrane. On their own, 70 nm cerasomes exhibited excellent transfection efficiency as was shown in [272].

The cerasome concept has been alternatively developed by Sebyakin and Gileva with co-workers who developed an L-aspartate-based cerasome forming lipids which were demonstrated to be able to form cerasomes comparable to the original cerasome forming lipids [273,274]. The aspartate-based framework enables simpler selection of hydrophobic tails, since in the original lipid structure the hydrophobic part is based on an amine that is more laborious to synthesize, while providing the opportunity to impart a cationic group in the structure to enable aqueous solubility as well. The silane coupling can be utilized for easy cerasome surface functionalization, as was done in [275], where the authors coupled TPP moiety to the silanol groups using APTES as a linker, forming a mitochondria-targeting cerasome formulation.

**Figure 20:**
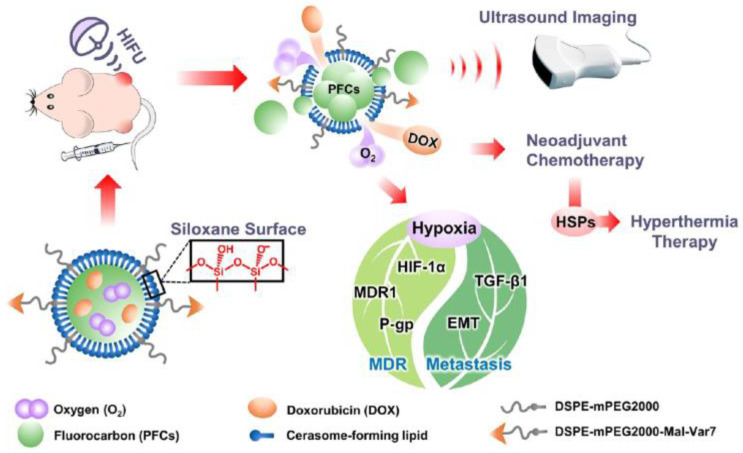
The schematic demonstration of a complex chemotherapeutic system. The cerasomes loaded with oxygen and DOX are delivered to tumor sites, where ultrasound irradiation helps release the cargo for dual action of treating hypoxia to prevent drug resistance and metastasis, and producing a cytotoxic effect caused by DOX for tumor mitigation. Reprinted with permission from [280]. Copyright 2020 American Chemical Society.

The CFL can be mixed with phospholipids, which produce a more permeable membrane to precisely tune release rates of any substrates, so, Dai et al. have compared a series of liposomal formulations based on different molar ratios of DPPC and CFL [276]. As a result, a series of hybrid cerasomal formulations which release the cargo at different rates were evaluated in terms of insulin delivery and the cerasome was able to sustain a hypoglycaemic effect in diabetic rats for 16 h. Another interesting effect that can be achieved by CFL mixtures with phospholipids is thermal responsiveness, which is based on the lipid phase transition temperature, as was shown in [277], where the authors obtained a formulation that released less than 20% of cargo at 37 °C within 90 min, but almost complete (80%) release was observed within the same period at 42 °C. Additionally, high-intensity focused ultrasound was widely applied for thermosensitive cerasomes, [278,279,280], showing a high potential of controlled localized drug release. Ultimately, utilizing the exceptional cerasome stability, researchers were able to encapsulate oxygen along with DOX to relieve tumor hypoxia for enhanced chemotherapeutic activity (Figure 20) [280].

It was shown that cerasomes can also be modified with contrast agents of radioactive and fluorescent nature for effective in vivo imaging [281,282], can be modified with oxidation sensitive fragments to create a redox-responsive nanocarrier [283], which can be externally triggered to release the cargo in the presence of cellular reducing environment.

Meanwhile along the biomedical application of cerasomes, many attempts are being made to develop novel functional materials using cerasomes as a framework. Song and co-authors constructed a cerasome coated with gold nanoparticles for horseradish peroxidase immobilization and electrocatalytical peroxide decomposition, which showed that cerasomes can be used as a biomimetic membrane to investigate mechanisms of various biological processes [284]. The same group used cerasome membrane as a catalyst for aerobic oxidation of thioglycol, and found that the metallophtalocyanine catalyst embedded into the cerasomes has higher catalytic activity than in traditional vesicles, micelles and organic solvents [285].

Overall, the cerasome emergence on the field of biomedical research is a significant development of the liposome concept. Cerasomes, the morphologically stable liposome analogues can bear all the benefits and modifications that are applicable to the liposomes, but they are also more stable during storage and in harsh conditions, are certainly required to develop novel ultimate drug formulations.

## 4. Conclusions

The review discusses the advantages of using modified liposomes as nanocarriers. Much attention is paid to the non-covalent modification of liposomes with cationic surfactants, which increases their stability, ability to overcome biological barriers, as well as ensure targeted drug delivery. In addition, non-covalent modification has a significant advantage over covalent modification in that there is no time-consuming multistep organic synthesis procedure. The problem of toxicity associated with cationic surfactants can be partially avoided by optimizing the composition of liposomes to compromise between the stability/targeting/permeability effects and toxicity profile.

The modification of liposomal drug delivery systems with peptides has taken place in research for more than 20 years. The peptides are usually applied to impart specific affinities of drug nanocarriers to certain target tissues, they are uniquely tweakable to be fit for any biomolecule and are indispensable assets in the design of modern drug delivery systems. However, they are not the only criterion of a promising nanocarrier, and many other techniques need to be applied to construct an all-round well-performing species, since liposomes are also prone to opsonization and RES uptake, decomposition in biological medium due to proteins, enzymes, oxidizers and other reactive species, aggregation during storage and other problems. Yet, some sort of targeting mechanics is necessary in any chemotherapeutic formulation, and for this purpose, there are countless peptide motifs fit for almost any target in the bioenvironment, with much more to be designed in the future.

Liposomes can also be modified with various macrocycles (cyclodextrins, porphyrins, calixarenes). Cyclodextrins, capable of forming guest–host complexes with hydrophobic drugs, improve the stability of their liposomal formulations. In this case, the hydrophobic drug is located in the cyclodextrin cavity rather than in the lipid bilayer, where its presence reduces the stability of the system. The inclusion of calixarenes with groups capable to bind to phospholipids or with charged functional groups also enhances the stability of liposomal formulations. The disadvantages of porphyrins, widely used in PDT and SDT, are eliminated by using them in the form of lipid formulations. Porphyrins loaded into liposomes exhibit higher toxicity to cancer cells, prevent tumor growth and also contribute to its shrinkage, prevent metastasis and reduce toxicity to healthy cells under irradiation. Hence, all the presented examples clearly demonstrate the high interest and potential of non-covalently modified lipid-based nanocarriers.

As an excellent inorganic alternative, mesoporous silica nanoparticles can be considered. Highly developed internal surface area allows them to encapsulate higher amounts of cargo compared to other delivery systems. Toxicity of silica nanoparticles can be controlled by their size and morphology, which may serve as the tool for avoiding of undesired side effects upon their accumulation and metabolism. However, real potential of porous drug carriers on the basis of silica is disclosed upon their various modification. Conjugation of silica matrixes with polymers or their envelopment it into lipid shell may provide versatile characteristics, stimuli-responsive properties (pH-responsive, thermosensitive, etc.), biocompatibility, thereby moving them towards clinical trials. Among the challenges in this field, development of highly ordered mesoporous materials with narrow size distribution should be mentioned. Upcoming investigations are predicted to address these points.

The combination of an inorganic silicon-containing component and an organic lipid component has led to the creation of another class of nanocarriers, cerasomes. Nowadays multiple research groups from different countries have significantly contributed to the development of the cerasome concept. The simple idea of covalently linked bilayer of hybrid amphiphilic monomers is a great and promising tool for the design of better and newer drug nanoformulations that combine beneficial features of both organic and inorganic carriers. Hopefully, more and more researchers would incorporate cerasomes into their biomedical work and further improve the already promising drug delivery platform.

## Figures and Tables

**Table 1:** Recent work examples (5 years to date) involving peptide-modified liposomes listed with corresponding peptides, targets or purposes, and the methods of liposome functionalization.

Ref	Peptides	Targets and Special Properties	Incorporation Mechanism	Object
[70]	^D^CDX (^D^G^D^R^D^E^D^I^D^R^D^TG^D^R^D^A^D^E^D^R^D^W^D^S-^D^E^D^K^D^F)	nAChR α7, overcoming the BBB	Thin film, DSPE-PEG anchor	U87 and mouse brain microvascular endothelial cells bEnd.3
[100,101]	APRPG	VEGFR-1, choroid treatment	Carbodiimide mediated conjugation to carboxyl on lipids to prepared liposomes	C57BL/6J mice, eye
[80]	DP7-C (VQWRIRVAVIRK)	Dendritic cells, immune adjuvant	Hydration with aqueous CHO-anchored peptide solution	293T and DC2.4, JAWSII and bone marrow-derived dendritic cells C57BL/6 J mice
[87]	cRGD	α_v_β_3_ and α_v_β_5_ integrins, localization in lung, brain, breast cancers	Thin film, DSPE-PEG anchor	MDA-MB-231 breast cancer cells
[66]	PFVYLI and R_9_F_2_ and transferrin	Enhanced internalization and targeting brain capillary endothelial cells.	Thin film, DSPE-PEG anchor	1-day-old rat brain cell cultures, Sprague Dawley rats, C57BL/6 mice
[89]	mn (mnRwr)	α_v_β_3_ integrin, localization in glioblastoma	Thin film, DSPE-PEG anchor	HUVEC, U87 MG, HL7702, HEK293 cells, BALB/c nude and ICR mice
[79]	CB5005 (KLKLAALALA-VQRKRQKLMP)	NF-κB (Nuclear factor-kB), a mechanism that suppresses apoptosis, to overcome irinotecan resistance	Thin film, DSPE-PEG anchor and Ethanol sol injection	A549, male nude mice
[91]	Pep-1 (KETWWETWWTEWSQPKKKRK-VC)	Enhanced cellular uptake	Thiol-maleimide conjugation to prepared liposomes	5637 and MBT2 bladder cancer cell lines
[71]	D8 (^D^RTG^D^R^D^A^D^RE^D^W)	nAChR (nicotinic acetylcholine receptor), overcoming the BBB	Thin film, DSPE-PEG anchor	Male SD rats, ICR mice, BALB/c nude mice
[92]	RIPL (IPLVVPLRRRRR-RRRC)	Enhanced cellular uptake, localization in hepsin overexpressing tissue	Thiol-maleimide conjugation to prepared liposomes	SK-OV-3 cells
[67]	THP (WNLPWYYSVSPTC)	HER2 (Human epidermal growth factor receptor-2), localization in breast, ovary, gastric, prostate cancers	Thin film, CHO anchor	MDA-MB453 cells
[86]	^D^T7 (^D^(HAIYPRH))	Transferrin receptor	Post-insertion	Fresh mouse serum HepG2, A549 and SK-OV-3 cells BALB/c nude mice
[78]	TAT-NBD (YGRKKRRQRRRGTTL-DWSWLQMEC)	NF-κB (Nuclear factor-kB), a mechanism that suppresses apoptosis, to overcome drug resistance	Thin film, DSPE-PEG anchor	4T1, RAW264.7, HUVEC cells Female BALB/c nude mice
[102]	(RL)_4_G-NH_2_	Enhanced cellular uptake	Post-insertion	HEK cells
[90]	P1c (CIRTPKISKPIKFELSG)	α_v_β_3_ integrin, localization in liver	Post-insertion, DSPE-PEG anchor	U87MG and MCF-7 cells
[82]	pHLIP ((ACEQNP-IWARYADWLFTTPLLLLDLALLV-DADEGT)	Low pH, localization in acidic media (tumors)	Thin film, DSPE-PEG anchor	Thorough liposome characterization
[103]	BP100 (KKLFKKILKYL-NH_2_), BP100-Ala-NH-C_16_H_33_, Cyclo(1-4)-cILC-BP100	Antibacterial action	Thin film	*Escherichia coli, Staphylococcus aureus, Bacillus subtilis*
[93]	TD (ACSSSPSKHCG)	Transcellular permeation by opening the paracellular pathway, melanoma treatment	Thin film, DSPE-PEG anchor	A375, B16F10, HUVEC cells BALB/c nude mice
[72]	Angiopep-2, T7, Peptide-22, c(RGDfK), D-SP5 and Pep-1	LRP1 (lipoprotein receptor-related protein-1), TfR (Transferrin receptor), Low-density lipoprotein receptor, α_v_β_3_ and α_v_β_5_ integrins, IL-13Rα2, overcoming the BBB/BBTB	Thin film, DSPE-PEG anchor	BCEC and HUVEC cells Intracranial glioma bearing mice
[88]	cRGD, _D_-(KLAKLAK)_2_	Localization in tumors and vascular endothelial cells and mitochondria targeting	Thin film, DSPE-PEG anchor	4T1 and HUVEC cells BALB/c mice
[104]	GNRQRWFVVWLGSTN- DPV-propargylglycine	Fibronectin, localization in bladder	Thin film, DSPE-PEG anchor	MB49 cells
[105]	ASSHNGC	Tumor vessels	Maleimide conjugation	hEPC, B16BL6 and Colon26 NL-17 cells C57BL/6 and BALB/c mice
[73]	Cys-R_4_, Cys-R_4_-dGR, Cys-R_6_ and Cys-R_6_-dGR	α_v_β_3_ integrin, neuropilin-1	Thin film, DSPE-PEG anchor	C6 glioma cells, tumor spheroids, Xenograft tumor-bearing BALB/c mice
[68]	KCC (KCCYSL)	HER2 (Human epidermal growth factor receptor-2), localization in breast	Thin film, DSPE-PEG and DSPE-[Serine-Glycine]_3,5,7_ anchors	SK-BR-3 and MDA-MB-231 breast cancer cells
[106]	L1 (EKEKEK-EPPPPGG)	Protection from protein adsorption for stealth-like properties	Direct hydration, freeze-thaw cycles and extrusion. Dipalmitoyl anchor	MIA PaCa-2 cells
	DW4 (Transferrin receptor DNA aptamer)	Transferrin receptor, cancer cellular uptake	Thiol-maleimide conjugation to prepared liposomes	
[83]	[D]-H_6_L_9_ and RGD	Low pH medium and α_v_β_3_ integrin	Thin film, DSPE-PEG anchor	C26 and MCF-7 cells C26 tumor bearing BALB/c mice
[96]	H_7_K(R_2_)_2_	Cell penetration, low pH responsiveness, localization in glioma	Thin fil, DSPE-PEG anchor	C6 and U87 MG cells C6 bearing BALB/c mice
[107]	L(R/K)QZZZL (Z-hydrophobic amino acids)	Transcytosis, overcoming the BBB	Thin film, DSPE-PEG anchor	MBEC4 cells
[76]	E_4_ [(EIAALEK)_4_] and K_4_ [(KIAALKE)_4_]	Membrane fusion for endocytosis	Thin film, CHO-PEG anchor	HeLa-K and HeLa-M cells Zebrafish HeLa xenograft
[94]	PSP (CGRRMKWKK-1-(bromomethyl)-4,5-dimethoxy-2-nitrobenzene), NGR (CYGGRGNG)	Enhanced cellular uptake	Post insertion, DSPE-PEG anchor	HT-1080, MCF-7 cells BALB/c mice
[95]	RF (GLKKLARLFHKLLKLGC)	Enhanced cellular uptake	Maleimide conjugation to prepared liposomes	PC-9, bEnd.3 cells
[81]	DP7-C (Chol-suc-VQWRIRVAVIRK-NH2)	Enhanced cellular uptake, antibacterial activity	Hydration with a CHO-conjugated peptide solution	HEK293 and LO2 cells BALB/c mice with MRSA-infectious murine model (methicillin-resistant *Staphylococcus aureus*)
[69]	c7AR	VEGFR-2 and neuropilin-1, localization in glioma	Thin film, DSPE-PEG anchor	HUVEC and U87 cells Matrigel based model SD rats, U87 xenograft bearing BALB/c mice
[108]	YSA (H_6_-PEG-YSAYPDSVPMMS)	EphA2 (ephrin type-A receptor 2), localization in lung	Post insertion, H_6_ anchor	A549 cells Nu/nu mice
[109]	OVA-I (SIINFEKL), and OVA-II (PSISQAVHAAHAEIN-EAPβA)	MHC (major histocompatibility complex) and dendritic cells	Post insertion	DC2.4, EL4, E.G7-OVA cells Female C57BL/6 mice

**Table 2:** Overview of the most studied types of MSN and characteristics of their structure, pore size, volume and surface area. Reprinted with permission from [169]. Copyright 2020 Elsevier.

MSN Type	Material Structure	Pore Size (nm)	Pore Volume (cm^3^/g)	Surface Area (m^2^/g)
MCM-41	2D hexagonal p6mm	1.6–10	0.7–1.2	1000
MCM-48	Bicontinuous cubic Ia3d	3.3	0.53–0.80	660–1010
SBA-3	2D hexagonal p6mm	2.6	0.98	1430
SBA-15	2D hexagonal p6mm	4.6–10	0.56–1.38	630–1040
SBA-16	Cubic Im3m	4.74–5.60	0.37–0.61	660–939
COK-12	2D hexagonal p6m	5.8–9.4	0.45–0.88	429–547
FDU-12	Face centered cubic fm3m	10.2	0.60–0.68	654–716
KIT-6	Bicontinuous cubic Ia3d	6.0–7.9	1.12–1.27	474–814

**Table 3:** Comparison of loading of various drugs in unfunctionalized and functionalized MSNs of different types. Reprinted with permission from [174]. Copyright 2018 MDPI.

Carrier	Drug	Loading (wt %)
MCM-41 HMSNs *	Ibuprofen	35.9 74.5
MCM-41 HMSNs	DOX	48.2 112.1
HMSNs HMSNs-NH_2_ * HMSNs-COOH * HMSNs-CN * HMSNs-CH_3_ *	5-fluorouracil	18.5 28.9 20.7 22.5 12.1
MCM-41_(C12)_ * MCM-41_(C16)_ * SBA-15	Captopril	23.6 34.0 22.6
MCM-41 SBA-15 SBA-15_(C8)_ * SBA-15_(C18)_ *	Erythromycin	29.0 34.0 13.0 18.0
MCM-41 MCM-41-NH_2_ * SBA-15 SBA-15-NH_2_ *	Alendronate	14.0 37.0 8.0 22.0
MSN-C0 MSN-C10	Lysozyme	34 42

* HMSN—Hollow mesoporous silica nanoparticles; abbreviations of functional groups used for modification of MSNs: C0—the absence of any modifying group, C8, C12, C16, C18—octyl, dodecyl, hexadecyl and octadecyl alkyl tails, respectively; NH_2_—amino group; CN—nitrile group; CH_3_—methyl group.

## Abbreviations

PEG	polyethylene glycol
PC	L-α-phosphatidylcholine
CHO	cholesterol
SHP	quaternary ammonium salts with a sterically hindered phenolic moiety
AChE	acetylcholinesterase
BBB	blood-brain barrier
2-PAM	pralidoxime chloride
TPP	triphenylphosphonium
Im	imidazolium
DPPC	1,2-dipalmitoyl-sn-glycero-3-phosphocholine
DOX	doxorubicin hydrochloride
HepG2	hepatocellular carcinoma
MTAB	(11-mercaptoundecyl)-N,N,N-trimethylammonium bromide
DSPE	1,2-Distearoyl-sn-glycero-3-phosphorylethanolamine
CPP	cell-penetrating peptide
VEGFR	vascular endothelial growth factor receptor
HER2	human epidermal growth factor receptor-2
nAChR α7	nicotinic acetylcholine receptor α7
IL-13Rα2	Interleukin-13 receptor subunit alpha-2
NBD	NF-κB essential modulator binding domain
BBTB	blood-brain tumor barrier
CD	cyclodextrin
HP-β-CD	2-hydroxypropyl-β-CD
M-β-CD	methyl-β-CD
SMR	sulfamerazine
INM	Indomethacin
DLS	dynamic light scattering
CUR	curcumin
DCL	drug-in-CD-in-liposome
ANE	anethole
ACL	anethole-in-CD-in-liposome
ACL2	anethole-double-loaded liposome
Quer	quercetin
PTX	paclitaxel
DLPL	double loaded PEGylated liposome
mTHPC	meta-tetrakis(3-hydroxyphenyl)chlorin
TM-β-CD	heptakis(2,3,6-tri-O-methyl)-β-CD
E4	estetrol
HDA-β-CD	heptakis(2,3-di-O-acetyl)-β-CD
POPC	1-palmitoyl-2-oleoyl-sn-glycero-3-phosphocholine
SC4AB	p-sulfonatocalix[4]arene tetrabutyl ether
SC4AH	p-sulfonatocalix[4]arene tetrahexyl ether
FITCPy	fluorescein isothiocyanate-conjugated pyridinium
PDT	photodynamic therapy
p-OH	5,10,15,20-tetrakis(4-hydroxyphenyl)porphyrin
SOPC	1-stearoyl-2-oleoyl-sn-glycero-3-phosphocholine
DOPC	1,2-dioleoylsn-glycero-3-phosphocholine
SLPC	1-stearoyl-2-linoleoyl-sn-glycero-3-phosphocholine
PPa	pyropheophorbide acid
Ce6	chlorin e6
LCP	low-molecular citrus pectin
DVDMS	sinoporphyrin sodium
DSPE-PEG 2000	1,2-distearoyl-sn-glycero-phosphoethanolamine-N-[methoxy(polyethyleneglycol)-2000]
TTMAPP	5,10,15,20-tetrakis(4-trimethylammoniophenyl)porphyrin
HMME	hematoporphyrin monomethyl ether
SDT	sonodynamic therapy
TTP	5,10,15,20-tetra-p-tolylporphyrin
*h*Ce6	hydrophobic Ce6
DiR	1,1′-dioctadecyl-3,3,30, 3′-tetramethylindotricarbocyanine iodide
TPZ	tirapazamine
ICG	indocyanine green
PEG-NI	2-nitroimidazole derivative linked to PEG
ECM	extracellular matrix
NCP	nanoscale coordination polymer
HUVEC	human umbilical vein endothelial cell
TPPS_4_	meso-tetrakis(4-sulfonatophenyl) porphine
DOTAP	1,2-dioleoyl-3-trimethylammonium-propane
GEM	gemcitabine hydrochloride
PDA	polydopamine
MSN	mesoporous silica nanoparticles
MCM	Mobil Crystalline Materials/Mobil Composition of Matter
SBA	Santa Barbara Amorphous
FDU	Fudan University Material
KIT	Korean Technological University
HMSN	hollow mesoporous silica nanoparticle
PV	pore volume
*S*	surface area
APTES	(3-aminopropyl)triethoxysilane
AuNP	gold nanoparticles
PNIPAM	poly-N-isopropylacrylamide
PUA	polyurethaneamine
PPG	polypropyleneglycol
GAP	azobenzene/galactose-grafted polymer
PEI	polyethyleneimine
PAMAM	poly(amido)amine
DOPE	1,2-dioleoyl-*sn*-glycero-3-phosphoethanolamine
DSPC	1,2-distearoyl-*sn*-glycero-3-phosphocholine
AZD1080	2-hydroxy-3-(5-(morpholinomethyl)pyridin-2-yl)-1*H*-indole-5-carbonitrile
CFL	cerasome-forming lipid
